# Subsampling Technique to Estimate Variance Component for UK-Biobank Traits

**DOI:** 10.3389/fgene.2021.612045

**Published:** 2021-03-05

**Authors:** Ting Xu, Guo-An Qi, Jun Zhu, Hai-Ming Xu, Guo-Bo Chen

**Affiliations:** ^1^Department of Mathematics, Zhejiang University, Hangzhou, China; ^2^Department of Agricultural and Biotechnology, Zhejiang University, Hangzhou, China; ^3^Zhejiang Provincial People's Hospital, People's Hospital of Hangzhou Medical College, Clinical Research Institute, Hangzhou, China; ^4^Key Laboratory of Endocrine Gland Diseases of Zhejiang Province, Hangzhou, China

**Keywords:** polygenicity, UK Biobank, subsampling estimator, effective number of markers, Haseman-Elston regression

## Abstract

The estimation of heritability has been an important question in statistical genetics. Due to the clear mathematical properties, the modified Haseman–Elston regression has been found a bridge that connects and develops various parallel heritability estimation methods. With the increasing sample size, estimating heritability for biobank-scale data poses a challenge for statistical computation, in particular that the calculation of the genetic relationship matrix is a huge challenge in statistical computation. Using the Haseman–Elston framework, in this study we explicitly analyzed the mathematical structure of the key term *tr*(***K***^*T*^***K***), the trace of high-order term of the genetic relationship matrix, a component involved in the estimation procedure. In this study, we proposed two estimators, which can estimate *tr*(***K***^*T*^***K***) with greatly reduced sampling variance compared to the existing method under the same computational complexity. We applied this method to 81 traits in UK Biobank data and compared the chromosome-wise partition heritability with the whole-genome heritability, also as an approach for testing polygenicity.

## Introduction

Given the increasing sample size and sequencing capability, high-throughput genetic data is presented as the standard input that challenges statistical computation. For example, in the estimation of heritability for complex traits using all markers concurrently, both (i) constructing the genetic relationship matrix [GRM, denoted as ***K*** and the mathematical expression can be seen in section Materials and Methods, with its computational cost O(*MN*^2^)] and (ii) the estimation of heritability using linear mixed model [O(*N*^3^)] are computationally expensive (Yang et al., 2010). In order to alleviate computational burden, various solutions have been proposed. Modified Haseman–Elston regression (HE) can be used to estimate heritability with reduced computational cost in the estimation step [O(*N*^2^)], but the construction of GRM is still needed (Chen, 2014). Using summary statistics, such as those estimated from the genome-wide association study (GWAS), rather than individual-level data, can provide a theoretical equivalence estimate of the heritability under the assumption that the source of summary statistics and the linkage disequilibrium (LD) reference are homogeneous (Bulik-Sullivan et al., 2015), if not always the case.

Even under the HE framework, given the availability of biobank-scale data, such as UK Biobank (UKB) data (Bycroft et al., 2018), the computational cost for GRM poses a challenge for heritability estimation mentioned procedure above. In order to reduce the computational cost of GRM, recently a randomized estimation of heritability has been introduced by Wu and Sankararaman (2018), called randomized Haseman–Elston regression (RHE), a promising method that can be used for both single-trait and bi-trait analyses (Sankararaman, 2019). This method is built on a hybrid framework which can be applied to biobank-scale data, and a key innovation involved is a quick evaluation for *tr*(***K***^*T*^***K***), the trace of the multiplication of GRM with its transpose. Direct computation of *tr*(***K***^*T*^***K***) can be time-consuming, at the time cost of O(*N*^2^*M*), but in RHE the numerical evaluation of *tr*(***K***^*T*^***K***) can be realized via a randomization method expressed in quadric form. However, we found that the sampling variance of RHE in the original report is incorrect because of their wrong derivation (refer to Appendix A3 in Wu and Sankararaman's original report). In this study, we further investigate the statistical property of RHE, in particular the term about *tr*(***K***^*T*^***K***), and its relevant extensions.

This report was written for three purposes. First, we found that the provided ***randomization*** ***estimate***for *tr*(***K***^*T*^***K***) is correct but with its sampling variance, which is proportional to *tr*(***K***^*T*^***KK***^*T*^***K***), not properly treated in Wu and Sankararaman's original report. We derived and numerically validated the sampling variance of *tr*(***K***^*T*^***K***). Second, recently a hybrid routine that can use either individual-level data and summary statistics has also been found (Zhou, 2017; Wu and Sankararaman, 2018), in which ***subsampling technique***is used to evaluate *tr*(***K***^*T*^***K***); however, its sampling variance was not available. We provided a similar method as subsampling but with availability of its analytical sampling variance. Third, we partitioned the heritability based on the ***effective number of markers***and applied them in the partitioning of heritability for some complex traits in UKB.

## Materials and Methods

### Genetic Relationship Matrix

For a homogenous unrelated sample, its genotypic matrix can be written as ***X***, a matrix of *N* rows—individuals, and *M* columns—coding the count of the reference allele for a biallelic locus. After standardization for each genotype ${\tilde{x}}_{kl}=\frac{x_{kl}-2p_{l}}{\surd 2p_{l}q_{l}}$, in which 2*p*_*l*_ is the allele frequency and $\sqrt{2p_{l}q_{l}}$ the square root of the variance, we can define GRM as $K=\frac{1}{M}\tilde{X}{\tilde{X}}^{T}$. Given ***K***, we can easily derive some characters of ***K***. Denote ***K***_*o*_ as the off-diagonal elements, and it is easy to see that $𝔼\;\left(K_{o}\right)=\frac{-1}{N-1}$, because the summation of the diagonal is *N* − 1. *var* (***K***_*o*_) is the sampling variance of the all off-diagonal elements.

Of note, *var* (***K***_*o*_) relates to the concept, so-called ***effective number of markers***, denoted as *M*_*e*_ thereafter. As noticed, *M*_*e*_ is defined as the reciprocal of *var* (***K***_*o*_). $M_{e}=\frac{1}{var\left(K_{o}\right)}=\frac{M^{2}}{M+\overset{M}{\underset{l_{1}\neq l_{2}}{\sum }}E{\left(\rho_{l_{1}l_{2}}\right)}^{2}}$, in which 𝔼 (ρ_*l*_1_*l*_2__) is the expected Pearson's correlation between the $l_{1}^{th}$ and $l_{2}^{th}$ loci. Alternatively, $M_{e}=\frac{1}{E{\left({\bar{\rho }}_{l_{1}l_{2}}\right)}^{2}}$. It is known that for a population, the averaged linkage disequilibrium across the genome is nearly a constant given the markers; in other words, *M*_*e*_ is a constant genetic parameter. The definition of *M*_*e*_ in this report allows researchers to calculate *M*_*e*_ based on a reference population of the same origin to the population in question. Similarly, *M*_*e*.*c*_ represents the averaged LD for any pair of markers on the *c*^*th*^ chromosomes.

As the causal variants are hardly observed directly, their relationship with markers are surrogated by relationship between markers, as reflected in *M*_*e*_. As *M*_*e*_ is a critical parameter in many genetic applications, a conceptional parameter is its involvement in genetic prediction (Dudbridge and Wray, 2013), or power calculation for the estimation of heritability (Visscher et al., 2014). In the estimation for variance components, as shown below, *M*_*e*_ is a key parameter.

### Haseman–Elston Regression Framework for the Estimation of Heritability

Haseman–Elston regression (HE) has been initially proposed for the linkage analysis (Haseman and Elston, 1972). With its original kernel relatedness between sib pairs via linkage replaced by linkage disequilibrium for unrelated samples, the modified HE can be used for the estimation of heritability (Chen, 2014). Due to its clear mathematical property, HE has been found a bridge to connect and develop various parallel methods for the estimation of heritability, such as LD score regression that estimates heritability and uses summary statistics from GWAS (Bulik-Sullivan et al., 2015; Zhou, 2017).

However, LD score regression is based on various assumptions that may or may not be met in practice. LD score regression uses SNPs in a sliding window instead of all genome-wide SNPs to calculate LD scores, which will lose efficiency if heterogeneity exists between the reference population and the population that generates the GWAS summary statistics. If we directly use individual-level data, the time cost will be unaffordable, such as for the restricted maximum likelihood estimation method (REML); in contrast, a method of moment (MoM) can provide equivalent estimation for the heritability for complex traits.

We assume that

$$\begin{array}{l} y=\tilde{X}\beta +e;\;\beta \sim N\left(0,\frac{h^{2}}{M}I\right);\;e\;\sim N\left(0,\sigma_{e}^{2}I\right) \end{array}$$

in which ***y*** is the standardized phenotype for a trait of interest, $\tilde{X}$ is the standardized genotypic matrix of *N* individuals and *M* the biallelic markers, *β* is the additive effect associated with each marker, ***e*** is the residual, *h*^2^ is the SNP heritability, and $\sigma_{e}^{2}$ is the residual variance. It is easy to know that $var\;\left(y\right)=𝔼\;\left(yy^{T}\right)-𝔼\;\left(y\right)𝔼\;\left(y^{T}\right)=\;\frac{h^{2}}{M}\tilde{X}{\tilde{X}}^{T}+\sigma_{e}^{2}I=h^{2}K+\sigma_{e}^{2}I$.

### Estimation for Heritability *via* Modified Randomized Haseman–Elston Regression

Consequently, we extend the work by Wu and Sankararaman (2018); the moment estimator is to minimize

$$\begin{array}{l} Q=tr\left\{{\left[yy^{T}-\left(h^{2}K+\sigma_{e}^{2}I\right)\right]}^{2}\right\} \end{array}$$

By taking the differentiation in terms of *h*^2^ and $\sigma_{e}^{2}$, we have

$$\left\{ \begin{array}{l} \frac{\partial Q}{\partial h^{2}}=tr\left\{h^{2}K^{T}K+\sigma_{e}^{2}K-yy^{T}K\right\}=0\\ \;\frac{\partial Q}{\partial \sigma_{e}^{2}}=tr\left\{h^{2}K+\sigma_{e}^{2}I-yy^{T}I\right\}=0 \end{array}\right.$$

After algebra, we have the normal equations below:

(1) $$\begin{array}{r} \left[\begin{array}{cc} tr\left(K^{T}K\right) & tr\left(K\right)\\ tr\left(K\right) & N \end{array}\right]\left[\begin{array}{c} {ĥ}^{2}\\ {\hat{\sigma }}_{e}^{2} \end{array}\right]=\left[\begin{array}{c} y^{T}Ky\\ y^{T}Iy \end{array}\right] \end{array}$$

The estimator for *ĥ*^2^ can be written as

(2) $$\begin{array}{r} {ĥ}^{2}=\frac{y^{T}\left(K-I\right)y}{tr\left(K^{T}K\right)-N} \end{array}$$

However, different computational strategies deal with the computational expensive part for both the numerator and the denominator. In particular, for the numerator, ***y***^*T*^***Ky*** can be decomposed as $y^{T}\tilde{X}{\tilde{X}}^{T}y/M$, and $y^{T}\tilde{X}$, equal to ${\left({\tilde{X}}^{T}y\right)}^{T}$. Each element ${\tilde{X}}_{j}^{T}y$ of ${\tilde{X}}^{T}y$ is just the regression coefficient between the *j*th marker and ***y*** that can be computed via simple linear regression, or multivariate linear regression if covariates are included. It is easy to recognize that $y^{T}\tilde{X}{\tilde{X}}^{T}y/M$ follows $\chi_{1}^{2}$ after scaling by the sample size *N*, and a possible non-central parameter related to the heritability of the trait. Alternatively, we derive the mathematical expectation $𝔼\;\left(y^{T}Ky\right)=N𝔼\;\left(\chi_{1|h^{2}}^{2}\right)=N\left(1+Nh^{2}{\bar{r}}^{2}\right)$, in which ${\bar{r}}^{2}$ is the averaged LD score between a marker to a causal variants in LD.

The denominator involves the trace of ***K***^*T*^***K***, a high-order function for GRM. Alternatively, according to the property of the trace of a matrix, it can be calculated that $tr(K^{T}K)=\sum_{i,j}^{N}K_{i,j}^{2}$, a summation of the square of each element in ***K***. From the first glance, it seems inevitable to compute ***K***, the computational cost of which is $O\left(N^{2}M\right)$, a substantial cost given a large sample size, such as for UKB of about 500,000 samples (Bycroft et al., 2018). In order to have a proper estimate for *tr*(***K***^*T*^***K***) but reduce computation cost, three methods are proposed for estimating *tr*(***K***^*T*^***K***).

### Estimating tr(K^T^K)

We present three methods in estimating *tr*(***K***^*T*^***K***). Sampling method I has been proposed by Wu and Sankararaman, but we provide its correct sampling variance, which was incorrectly given in their original report (Wu and Sankararaman, 2018). Sampling method II derives the expectation of *tr*(***K***^*T*^***K***) and estimates it in a reference population with the similar genetic origin of the population of question. Sampling method III slightly modifies method II if the reference population is big and yields smaller sampling variance of *tr*(***K***^*T*^***K***) than that of method II.

### Sampling Method I: The Randomized Estimator With Corrected Analytical Sampling Variance

Using randomized estimation, an unbiased estimator *L*_*B*_ is employed to estimate *tr*(***K***^*T*^***K***) in RHE (Wu and Sankararaman, 2018). The rational for a randomized estimate is as below:

$$\begin{array}{l} L_{B}=\frac{1}{B}\frac{1}{M^{2}}\overset{B}{\underset{b}{\sum }}tr\left(z_{b}^{T}XX^{T}XX^{T}z_{b}\right)=tr\left(K^{T}K\right) \end{array}$$

In each iteration, a vector ***z***, of length *N*, is generated from the standard normal distribution. As long as ***z*** has been generated *B* time and *B* is large enough, it is guaranteed to approach *tr*(***K***^*T*^***K***). As $z_{b}^{T}XX^{T}$ can be calculated easily, the computational cost is O(NMB). Then, *L*_*B*_ can be plugged into a normal equation (Equation 2).

In Wu and Sankararaman's original report, the sampling variance of *L*_*B*_ was given as $var\left(L_{B}\right)=2tr\left(K^{T}K\right)/B$, which was incorrect, and the correct one should have been

(3) $$\begin{array}{l} var\left(L_{B}\right)\equiv Var\left(\frac{1}{B}\sum_{b=1}^{B}z_{b}^{T}K^{T}Kz_{b}\right)\\ \;=\frac{1}{B^{2}}\sum_{b=1}^{B}Var\left(z_{b}^{T}K^{T}Kz_{b}\right)\\ \;=\frac{1}{B^{2}}\sum_{b=1}^{B}2tr\left(K^{T}KK^{T}K\right)=\frac{2tr\left(K^{4}\right)}{B} \end{array}$$

The derivation of the penultimate step uses the quadratic variance calculation formula. The sampling variance of *L*_*B*_ is proportional to *tr*(***K***^*T*^***KK***^*T*^***K***), the computational cost of which is likely to be infeasible for biobank-scale data. However, its practical sampling variance can be estimated from *B* iterations above $var\;\left(L_{B}\right)={\overset{B}{\underset{j=1}{\sum }}\left(L_{B_{j}}-{\bar{L}}_{B}\;\right)}^{2}/B$.

### Sampling Method II: Estimating *tr*(*K*^*T*^*K*) by Subsampling

**Figure 1:**
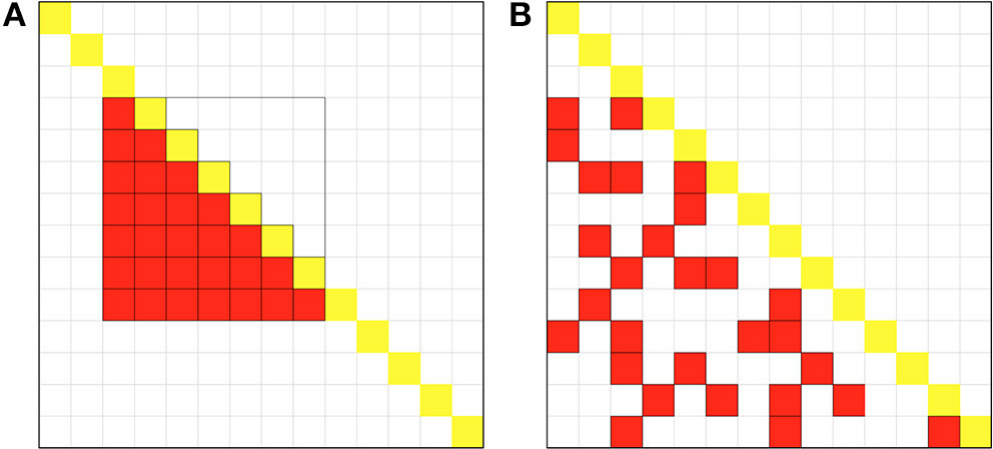
Schematic diagram of the sampling methods for the two estimators. The large square represents the entire genetic relationship matrix (GRM) with sample size *N* = 14, and each small an element in GRM, and the yellow the diagonal element of the matrix. **(A)** Schematic diagram of sampling methods for the *L*_*S*_ estimator. The red squares in the lower triangular of GRM represent the pairs of samples for *L*_*S*_. This figure shows that when *B* = 2 (the parameter for the *L*_*B*_ estimator given by Wu and Sankararaman, 2018), we set subsample size $s=\sqrt{2BN}\approx$ 7 for *L*_*S*_ to guarantee the same computational cost. **(B)** Schematic diagram of sampling methods for the *L*_*T*_ estimator. Equally total number (*n* = *BN* = 28) of GRM elements in the lower triangular in red represents the pair of the samples for *L*_*T*_. The elements are drawn as if they are randomly, as shot by a gun, to reduce correlation between individuals.

In an alternative route, we bypass the direct computation of *tr*(***K***^*T*^***K***). It is shown that $tr\left(K^{T}K\right)=N^{2}/M_{e}+N$ for unrelated samples (see Supplementary Notes). *N* is the sample size, a known parameter; we only need to estimate *M*_*e*_. As noted above, *M*_*e*_ can be estimated by subsampling a proportion of the study population (Figure 1) or by a reference population of the same origin with the population of study (Zhou, 2017). Thus, we can estimate *M*_*e*_ using a small proportion of the sample, as long as we can estimate ${\hat{M}}_{e}$; we can easily get the estimator of *tr*(***K***^*T*^***K***). We define a new *L*_*S*_ estimator: $L_{S}\equiv N^{2}/{\hat{M}}_{e}+N$. It is an unbiased estimate (see Supplementary Notes). Suppose the sample size of subsample is *s*, there are *s*^2^/2 off-diagonal elements and it takes O(*s*^2^*M*/2) time to calculate ${\hat{M}}_{e}$. The sampling variance of *L*_*S*_ is, using the Delta method, ${\left(L_{s}^{{}^{\prime }}\right)}^{2}var\left({\hat{M}}_{e}\right)=\frac{N^{4}}{M_{e}^{4}}\sigma_{{\hat{M}}_{e}}^{2}$, in which $L_{S}^{{}^{\prime }}=-\frac{N^{2}}{M_{e}^{2}}$ the first derivative of *L*_*S*_ and $\sigma_{{\hat{M}}_{e}}^{2}$ the sampling variance for ${\hat{M}}_{e}$. $\sigma_{{\hat{M}}_{e}}^{2}$ is not directly known but can be directly estimated in the third method proposed below.

### Sampling Method III: Estimating *tr*(*K*^*T*^*K*) *via* Shotgun Randomization

However, $\sigma_{{\hat{M}}_{e}}^{2}$ in method II is not analytical probably because each individual will be involved *s* times in the estimation of variance. With slightly modification, we developed a new estimator *L*_*T*_ (lower triangle shotgun sampling estimator) to estimate *tr*(***K***^*T*^***K***) in RHE. The difference in sampling schemes between methods II and III can be visualized as Figure 1. Given the whole GRM, method II samples a square matrix of size *s* × *s* after rearrangement and calculates half elements, whereas method III randomly samples *n* = *s*^2^/2 elements in the whole GRM without replacement so as to reduce overlapping of samples (Figure 1). This sampling idea is similar to the shotgun method in the first-generation DNA sequencing technology, so we call the method III shotgun sampling estimator.

Given a random subset of *n* elements *A* ⊆ {1, 2, ⋯ , *N*(*N* − 1)/2}, we define

$$\begin{array}{l} L_{T}\equiv N+\frac{N^{2}}{n}\sum_{i=1}^{n}{K_{o}}_{A_{i}}^{2} \end{array}$$

It can be proved that *L*_*T*_ is an unbiased estimator of *tr*(***K***^*T*^***K***) with its sampling variance $N^{4}var\left({K_{o}}^{2}\right)/n$, which can be estimated by $N^{4}var\left({K_{o}}_{A_{i}}^{2}\right)/n$ (see Supplementary Notes). Therefore, we can get the unbiased estimate of *tr*(***K***^*T*^***K***) and its sampling variance at the same time in one step. It does not need to calculate all the elements in ***K***_*o*_ but the corresponding pairs of the individuals, and to calculate the mean of the product of all their genetic values. Therefore, each item in the summation can be computed in O(*M*), and the total running time is O(*nM*).

### The Estimation of Variance Components and Its Sampling Variance

If we replace *tr* (***K***^*T*^***K***) with its subsampling estimators, we can get the synthesized estimator for heritability

$$\begin{array}{l} {ĥ}^{2}=\frac{N𝔼\;\left(\chi_{1|h^{2}}^{2}\right)-N}{N^{2}/{\hat{M}}_{e}}={\hat{M}}_{e}h^{2}{\bar{r}}^{2} \end{array}$$

where $𝔼\;\left(\chi_{1|h^{2}}^{2}\right)$ is the mean of $\chi_{1}^{2}$ for each SNP with or without the adjustment of covariates. Using the Delta method, we show in Supplementary Notes that the variance of *ĥ*^2^ can be formulated as

$$\begin{array}{l} \sigma_{h^{2}}\approx \frac{2M_{e}}{N^{2}}+{M_{e}}^{2}\frac{\sigma_{{K_{o}}^{2}}^{2}}{n}{\left(h^{2}\right)}^{2} \end{array}$$

and each item in the above formula is estimable, then we can get the variance estimator of the variance component

(4) $$\begin{array}{l} {\hat{\sigma }}_{h^{2}}=\frac{2{\hat{M}}_{e}}{N^{2}}+{{\hat{M}}_{e}}^{2}\frac{\sigma_{{K_{o_{A_{i}}}}^{2}}^{2}}{n}{\left({ĥ}^{2}\right)}^{2} \end{array}$$

Except for *ĥ*^2^, all other parts involved are independent to the phenotype, so given a specific sample of question, the estimator has a linear relationship with the square of the estimated heritability.

### Genetic Partitioning of Heritability

Yang et al. (2010) estimated the chromosome-wise partitioned heritability and found that the heritability of complex trait, such as human height is proportional to the length of the chromosome, that is, proportional to the number of causal variants. Some researchers gave more weight to large effects to explain heritability and to study polygenicity (O'Connor et al., 2019; Yang and Zhou, 2020). In this report, we instead calculated heritability based on ${\hat{M}}_{e}$ and compared the chromosome-wise partition heritability with the whole-genome heritability

(5) $$\begin{array}{l} {ĥ}_{C}^{2}=\overset{22}{\underset{c=1}{\sum }}\frac{N𝔼\;\left(\chi_{1|h^{2}}^{2}\right)-N}{N^{2}/{\hat{M}}_{e.c}}=\overset{22}{\underset{c=1}{\sum }}{\hat{M}}_{e.c}h_{c}^{2}{\bar{r}}_{c}^{2} \end{array}$$

in which ${\hat{M}}_{e.c}$ is the effective of markers for the *c*^*th*^ chromosome and ${\bar{r}}_{c}^{2}$ is the averaged squared correlation between a casual variant and a marker on the *c*^*th*^ chromosome. Under the assumption of polygenicity, ${ĥ}_{C}^{2}=\frac{h^{2}}{{\hat{M}}_{e}}\overset{22}{\underset{c=1}{\sum }}{\bar{r}}_{c}^{2}$, and the ratio between $\frac{h^{2}}{h_{C}^{2}}=\frac{{\bar{r}}^{2}}{\overset{22}{\underset{c=1}{\sum }}r_{C}^{2}}$. As both ${\bar{r}}^{2}$ and $\overset{22}{\underset{c=1}{\sum }}{\bar{r}}_{c}^{2}$ are unknown, we use $\frac{1}{M_{e}}$ and $\frac{1}{\overset{C}{\underset{c=1}{\sum }}M_{e.c}}$ as the surrogates for ${\bar{r}}^{2}$ and $\overset{22}{\underset{c=\;1}{\sum }}{\bar{r}}_{c}^{2}$.

By breaking the GRM of the whole genome ***K*** in Equation (1) into the GRMs for 22 autosomes, we can also estimate the chromosome heritability jointly in one model. This method has to inverse a 23 × 23 matrix. Under the assumption that the genotype of each chromosome contains the same *N* individuals, the inversed matrix is completely upon *N* and *M*_*e*.*c*_, so a computation cost linear to 23, without bothering the conventional matrix inversion procedure, a computation cost of 23^3^, can be written down analytically. In particular, the *c*^*th*^ diagonal element of the inverse matrix is $M_{e.c}/N^{2}$, and the last column/row is ${-M}_{e.c}/N^{2}$. For more details, please see Supplementary Notes.

### Heritability for the Weighted Genetic Relationship Matrix

Given the definition of the weighted GRM

$$K_{w}=\frac{\sum_{l=1}^{M}\left(x_{il}-2p_{l})(x_{jl}-2p_{l}\right)}{\sum_{l=1}^{M}2p_{l}q_{l}}$$

we can get an estimator of the weighted heritability as well as its variance estimator based on weighted GRM through a similar derivation

$$\begin{array}{l} {ĥ}_{w}^{2}=\frac{N\frac{\sum_{l=1}^{M}2p_{l}q_{l}\chi_{1|h^{2},l}^{2}}{\sum_{l=1}^{M}2p_{l}q_{l}}-N}{N^{2}/{\hat{M}}_{ew}},\;{\hat{\sigma }}_{h_{w}^{2}}=\frac{2{\hat{M}}_{ew}}{N^{2}}+{{\hat{M}}_{ew}}^{2}\frac{\sigma_{{K_{o_{A_{i}}}}^{2}}^{2}}{n}{\left({ĥ}_{w}^{2}\right)}^{2} \end{array}$$

where ${\hat{M}}_{ew}$ is the estimation of *M*_*e*_ for ***K***_*w*_, and $\chi_{1|h^{2},l}^{2}$ is the square of the z-score for the *l*^*th*^ SNP with or without the adjustment of covariates. The weighted chromosome-wise partition heritability can be expressed as

$$\begin{array}{l} {ĥ}_{Cw}^{2}=\overset{22}{\underset{c=1}{\sum }}\frac{N\frac{\sum_{l=1}^{M_{c}}2p_{l}q_{l}\chi_{1|h^{2},l}^{2}}{\sum_{l=1}^{M_{c}}2p_{l}q_{l}}-N}{N^{2}/{\hat{M}}_{ew.c}} \end{array}$$

where ${\hat{M}}_{ew.c}$ is the estimation of weighted *M*_*e*_ for the *c*^*th*^ chromosome and *M*_*c*_ is the number of SNP of the *c*^*th*^ chromosome.

### Connection to Other Estimators

The BOLT-LMM method (Loh et al., 2015) might be the most widely used method in the field of heritability estimation for large-scale data. Theoretically, the computational complexity of BOLT-LMM is $O\left(PMN^{1.5}\right)$, where *P* is the number of iterations for convergence. In the *L*_*T*_ estimator, the subsample size *n* ≪ *N*^1.5^, so our calculation time is less than BOLT-LMM in theory. In terms of actual calculation, the *L*_*B*_ estimator used less calculation time to get an accuracy similar to BOLT-LMM (Wu and Sankararaman, 2018); the variance of our estimators is about an order of magnitude smaller than *L*_*B*_ under the same calculation time. Thus, our method is better than BOLT-LMM in calculation accuracy and time. In terms of memory, the memory complexity of BOLT-LMM is O(*NM*/4), while the memory of our subsampling estimators is proportional to *M* and the subsample size *s* in the *L*_*S*_ estimator, which generally does not exceed 10% of the total sample size.

Given the availability of the estimators and their sampling variances, it is able to evaluate the statistical power of the estimators and estimate the sample size for the given type I and type II error rates. Under the null hypothesis *h*^2^ = 0, the sampling variance for the additive variance component can be reduced to ${\hat{\sigma }}_{h^{2}}\approx \frac{2{\hat{M}}_{e}}{N^{2}}$, which are equivalent to that of REML (Visscher et al., 2014). It is consequently known that the statistical power of the presented method will be equivalent to REML. In contrast, the original Haseman–Elston regression has doubled sampling variances where ${\hat{\sigma }}_{h^{2}}\approx \frac{4{\hat{M}}_{e}}{N^{2}}$ (Chen, 2014), because the original HE regression only uses the off/upper-diagonal of the matrix, as presented in the numerator above. The connection to LD score regression is obviously too; here, the whole *M*_*e*_ can be seen as a genome LD score, rather than being partitioned into genomic bins.

## Results

### Simulation Results for the Evaluation of *tr*(*K*^*T*^*K*)

**Figure 2:**
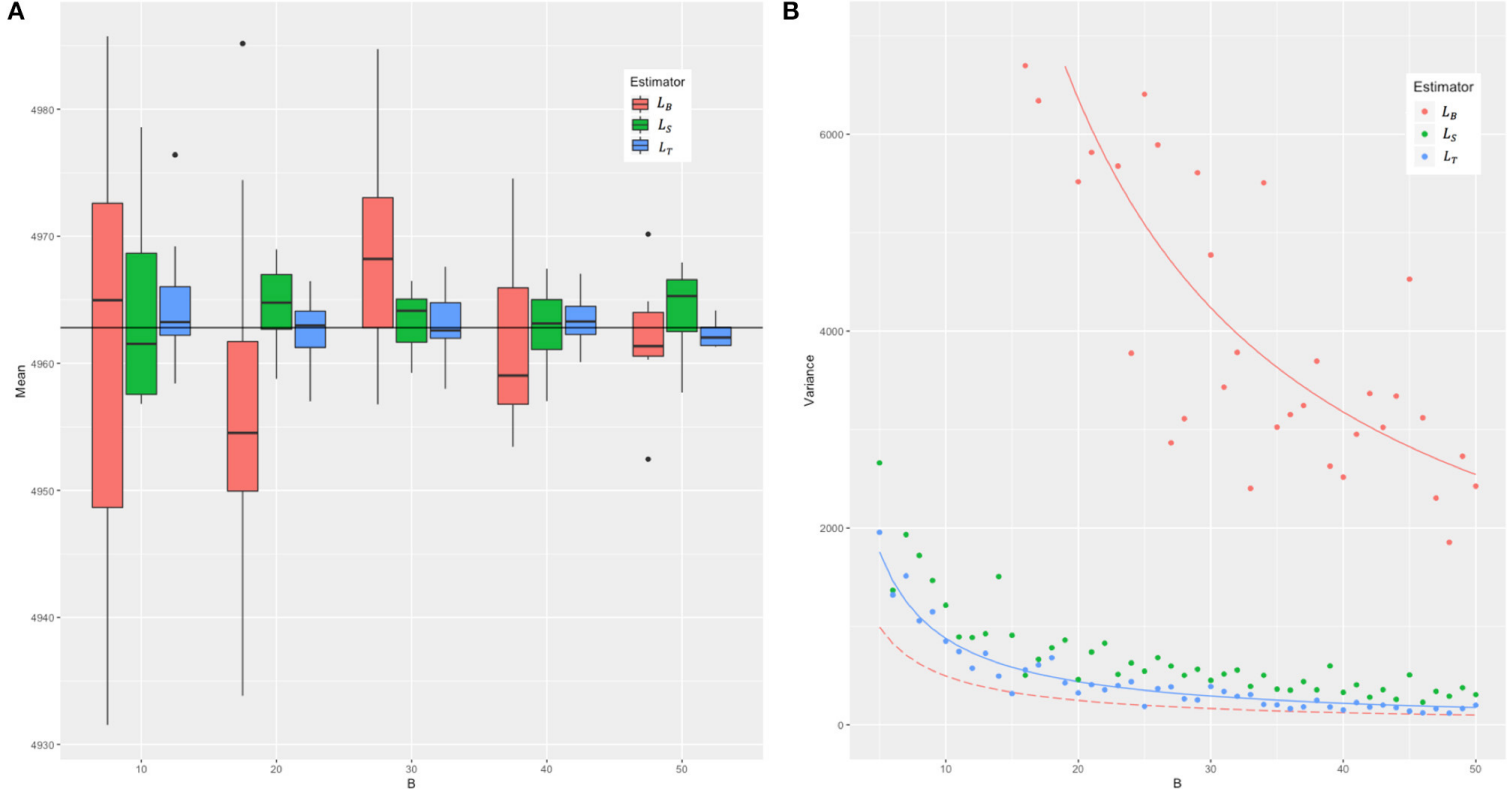
The means and the sampling variances of three estimators in simulation. The genotype data were constituted by 2,000 individuals and 2,000 markers with strong LD (from 0.6 to 0.8 in Lewontin's measure *D*′). *B* is the parameter related to the iteration for *L*_*B*_. For each *B*, the number of samples of the *L*_*S*_ and *L*_*T*_ estimators was adjusted to ensure that the three estimators have the same computational cost. **(A)** The boxplots of means and **(B)** the variance of 100 random experiments of three estimators for each *B*. In **(A)**, we combined 10 cases with *B* = 5–14 into the first group, and so on, and the last five cases with *B* = 46–50 were combined into the fifth group. The red boxes represent the means of *L*_*B*_, green the means of *L*_*S*_, and blue the means of *L*_*T*_, respectively. The black horizontal line represents the real value of *tr*(***K***^*T*^***K***). In **(B)**, the solid red line represents the true sampling variance of the *L*_*B*_ estimator derived in this study, and in contrast the long-dash red line the sampling variance of the *L*_*B*_ estimator incorrectly given by Wu and Sankararaman (2018). The solid blue line represents the theoretical sampling variance of *L*_*T*_. The solid green line is not given because we cannot get the theoretical sampling variance of *L*_*S*_ yet.

In the simulation and in the real data, we compared the mean and variance of the three estimators *L*_*B*_, *L*_*S*_, and *L*_*T*_, and the results are as presented in Figure 2. We took *n* = *BN* and $s=\sqrt{2BN}$ to make sure the three estimators are under the equal computational cost of O(*NMB*) (see Figure 1 for an example). In the simulation, we set the genotype in two ways: (1) The minor allele frequency (MAF) of each SNP was randomly generated from a uniform distribution between 0.03 and 0.5, and two levels of LD (linkage disequilibrium, in terms of Lewontin's *D*′, the normalized LD parameter) strength were set as 0–0.2 (weak LD) and 0.6–0.8 (strong LD) with the SNP number *M* = 2,000, 5,000 and sample size *N* = 500, 1,000, and 2,000, respectively. (2) The real genotype data consisted of 12,980 adjacent markers on chromosome 22 of 2,000 randomly sampled unrelated white British individuals in UKB. *B* was set from 5 to 50 and repeated 100 times for each to assess the mean and variance of the three estimators.

Across different parameter settings (sample sizes, number of loci, MAF, and LD), it yielded a similar pattern for the evaluated results of *tr*(***K***^*T*^***K***). We chose *M* = *N* = 2,000 and strong LD for detailed presentation, and the rest were shown in Supplementary Figures 1, 2). Figure 2 shows that all the three estimators were unbiased. The variance of each of these estimators, as expected, was inversely proportional to *B*. The real sampling variance of *L*_*B*_ was several times larger than the analytical incorrect result given in Wu and Sankararaman's study (refer to Appendix A3 in their original report) but was consistent with 2*tr* (***K***^*T*^***KK***^*T*^***K***)/*B*, just the corrected one as derived in this study (Equation 3). The sampling variance of *L*_*T*_ was about an order of magnitude smaller than that of *L*_*B*_. The simulation results in the real data shown in Supplementary Figure 3 were consistent with Figure 2.

### Real Data Analysis for *tr*(*K*^*T*^*K*)

We compared the performance of the three estimators *L*_*B*_, *L*_*S*_, and *L*_*T*_ in UKB. After quality control, 525,460 autosome SNPs with MAF > 0.01 for 278,788 unrelated British white individuals, whose pairwise genetic relationship coefficient <0.0125, were included for analysis. We set *B* = 5, 10, 20, 40, 60, 80, and 100, and calculated each of the three estimators 100 times to get the mean and the variance for each *B*. We compared the means of the three estimators with the expected value of $tr\left(K^{T}K\right)=N^{2}/{\hat{M}}_{e}+N$, where ${\hat{M}}_{e}$ was estimated from subsamples; given with ${\hat{M}}_{e}\approx \;$87,351, *tr* (***K***^*T*^***K***) was expected to be 1,168,573 for each of the three estimators.

The calculation was performed on an Intel(R) Xeon(R) Bronze 3104 CPU @ 1.70-GHz server cluster, and about 30 threads were allocated for each calculation. The actual calculation time of the three estimators were basically the same (see Supplementary Table 1) and conformed to the theoretical calculation complexity O(*NMB*). The variances of the three estimators *L*_*B*_, *L*_*S*_, and *L*_*T*_ for *tr*(***K***^*T*^***K***) are listed in Table 1. In particular, between the randomized estimator and the subsampling estimators, there was a huge difference between their variances. Under the real data, the sampling variance of *L*_*B*_ was large, while the sampling variances of the other two estimators were smaller and the variance of *L*_*T*_ was about half that of *L*_*S*_. The variances of each of the three estimators decreased with the increasing *B*, consistent with the simulation.

**Table 1:** The sampling variance of the three estimators.

**Estimator**	***B* = 5**	***B* = 10**	***B* = 20**	***B* = 40**	***B* = 60**	***B* = 80**	***B* = 100**
*L*_*B*_	13,918,476	6,970,471	3,501,313	1,689,667	1,214,649	875,951	781,079
*L*_*S*_	4,185,873	982,406	501,486	267,914	138,344	142,868	186,764
*L*_*T*_	1,587,955	787,997	389,442	217,566	130,818	8,6461	81,037

*B represents the iterations taken by L_B_. We took sample size $s=\sqrt{2BN}$ for L_S_ and n = BN for L_T_ in each step to guarantee the three estimators having the equal computational cost of O(*NMB*), where N is the total sample size*.

### Chromosome-Wise Partitioning for Heritability

Equation (2) presents how heritability is estimated using all 22 autosomes, and Equation (5) offers an alternative method by summation of chromosome-wise estimation for heritability. For ease of comparison, we only estimated heritability for 81 traits as demonstrated by Ge et al. (2017). We used the first two principal components as the covariates to control the possible population stratification; other covariates were adjusted upon the traits. The chromosome-wise partition heritability was calculated by the summation of the heritability estimated for *M*_*e*.*c*_ for each chromosome (Table 2), and the whole-genome heritability was calculated from the GRM of the whole genome.

**Table 2:** ${\hat{M}}_{e.c}$ and ${\hat{M}}_{ew.c}$ of each autosome.

**Autosome**	**Number of markers**	**${\hat{M}}_{e.c}$**	**${\hat{M}}_{ew.c}$**
1	41,805	10,333.95	5,531.40
2	42,087	10,131.61	5,410.58
3	35,488	8,377.99	4,557.51
4	33,248	8,168.11	4,567.46
5	31,855	7,772.11	4,200.29
6	36,643	1,217.21	522.52
7	28,868	6,996.85	3,882.00
8	27,244	5,878.64	2,941.71
9	23,120	6,172.40	3,423.64
10	26,242	5,978.38	3,607.96
11	26,119	4,978.77	2,835.29
12	25,041	6,204.85	3,385.99
13	18,065	4,988.62	2,802.15
14	17,040	4,492.59	2,458.88
15	16,555	3,911.11	2,174.81
16	18,570	4,448.96	2,461.84
17	17,140	3,868.71	2,040.32
18	15,837	4,561.48	2,549.61
19	13,998	3,151.14	1,816.42
20	13,997	3,800.48	2,080.39
21	7,949	2,223.92	1,231.52
22	8,549	2,114.08	1,240.90

*${\hat{M}}_{e.c}$ is the estimation of the effective of markers (*M*_*e*_) for the *c*^*th*^ autosome, and ${\hat{M}}_{ew.c}$ is the estimation of weighted *M*_*e*_ for the *c*^*th*^ autosome*.

The estimated heritability of some selected UKB traits is listed in Table 3 (see Supplementary Table 2 for all the 81 traits). The heritability of all traits was basically very similar to Ge et al.'s result and within the error range. Several physiological traits, such as height and weight had high heritability, while social traits that were more affected by social factors, such as the duration of certain activities, showed lower heritability. This result was consistent with the mainstream conclusion. The left part of Equation (4) for variance estimators of the whole-genome heritability ($\frac{2{\hat{M}}_{e}}{N^{2}}$) contributed a large part of the total variance (about 0.0017 in 0.002 for *N* = 270,000). Although the variances of the ${\hat{L}}_{B}$, ${\hat{L}}_{S}$, and ${\hat{L}}_{T}$ were several times different, they influenced little on the variance of estimated heritability.

**Table 3:** Estimation of heritability for some traits in UK Biobank.

**Field ID**	**Field name**	***N***	**${\hat{h}}_{Chr}^{2}$**	**${\hat{h}}_{Gen}^{2}$**	**${\hat{h}}_{se}^{2}$**
3786	Age asthma diagnosed	31,535	0.271	0.265	0.013
2754	Age at first live birth	100,951	0.281	0.217	0.004
2976	Age diabetes diagnosed	12,628	0.231	0.618	0.033
2139	Age first had sexual intercourse	255,880	0.064	0.051	0.002
21001	Body mass index (BMI)	277,223	0.360	0.282	0.002
4079	Diastolic blood pressure, automated reading	259,815	0.199	0.161	0.002
894	Duration of moderate activity	231,311	0.045	0.034	0.002
914	Duration of vigorous activity	166,696	0.032	0.025	0.003
874	Duration of walks	267,826	0.055	0.040	0.002
20150	Forced expiratory volume in 1-s (FEV1), best measure	207,848	0.257	0.207	0.002
20151	Forced vital capacity (FVC), best measure	207,848	0.314	0.252	0.002
2149	Lifetime number of sexual partners	253,460	0.011	0.008	0.002
20127	Neuroticism score	226,198	0.160	0.133	0.002
20161	Pack years of smoking	81,555	0.275	0.201	0.005
102	Pulse rate, automated reading	259,815	0.217	0.170	0.002
21021	Pulse wave arterial stiffness index	92,137	0.045	0.033	0.005
1299	Salad/raw vegetable intake	278,142	0.079	0.057	0.002
20015	Sitting height	277,231	0.528	0.444	0.002
1160	Sleep duration	278,142	0.089	0.070	0.002
50	Standing height	277,508	0.895	0.729	0.002
4080	Systolic blood pressure, automated reading	259,812	0.194	0.155	0.002
48	Waist circumference	277,649	0.278	0.219	0.002
1528	Water intake	278,142	0.098	0.075	0.002
21002	Weight	277,325	0.372	0.299	0.002
23102	Whole body water mass	273,248	0.468	0.386	0.002

*N is the sample size, ${\hat{h}}_{Chr}^{2}$ is the chromosome-wise partition heritability calculated by adding the heritability of each chromosome, ${\hat{h}}_{Gen}^{2}$ is the whole-genome heritability calculated from the GRM of the whole genome, and ${\hat{h}}_{se}^{2}$ is the standard error of the whole-genome heritability*.

**Figure 3:**
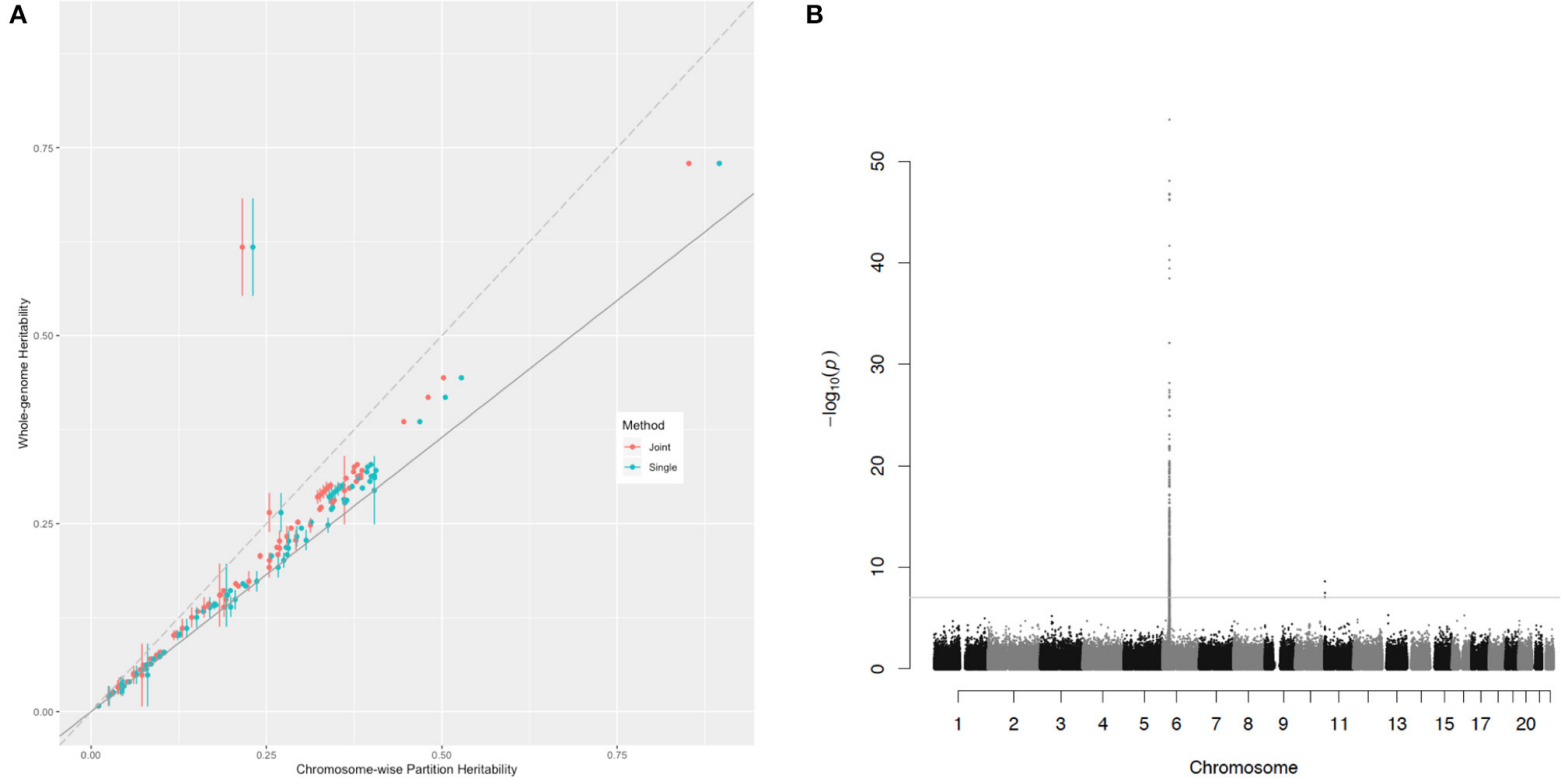
The relationship of the chromosome-wise partition heritability and the whole-genome heritability. **(A)** Each dot represents a trait in the UKB dataset listed in Supplementary Table 2. The horizontal axis represents their chromosome-wise partition heritability, and the vertical line at each point is their error bar; the vertical axis represents their whole-genome heritability. The red color represents the chromosome-wise partition heritability calculated jointly, and the green color represents the chromosome-wise partition heritability calculated singly. The long-dash line crosses the origin has a slope of 1. The slop of the solid line is 0.729, the ratio of $\frac{{\hat{M}}_{e}}{\overset{22}{\underset{c=1}{\sum }}{\hat{m}}_{e.c}}$. **(B)** The Manhattan plot for the trait age diabetes diagnosed, and the threshold is for genome-wise threshold for α= 0.05 after Bonferroni correction.

In the comparison of the two kinds of heritability for each trait, all the chromosome-wise partition heritability was higher than the whole-genome heritability except for the trait of the age diabetes diagnosed (the explanation of this exception is given below). For a certain polygenic trait, the heritability attributed to each chromosome was proportional to ${\hat{M}}_{e.c}$ according to the heritability estimation formula (Equation 5). Since the LD score between chromosomes could be considered as 0, this causes the ${\hat{M}}_{e}$ of the whole genome to be diluted by a large number of blank LD, so the overall ${\hat{M}}_{e}$ was smaller than the average ${\hat{M}}_{e.c}$ of each chromosome. In order to eliminate the influence of blank LD to see the contribution of effects of causal variants to heritability, Figure 3 shows the relationship of these two estimations of the heritability for the 81 traits. The slope of the solid gray line in the figure represents the ratio of the whole-genome ${\hat{M}}_{e}$ to $\overset{c}{\underset{i=1}{\sum }}{\hat{M}}_{e.c}$, a ratio of 0.729. This figure was to capture traits that do not meet the assumptions of polygenic assumption—or fitness of the model. If a trait were purely polygenic, the point representing this trait would be expected just along the solid gray line. However, the points were mostly distributed above the line, indicating that the effect size of causal variants was not evenly distributed on the chromosomes. In particular, the trait of the age diabetes was diagnosed, the Manhattan plot of which showed many statistically significant SNPs concentrated on the major histocompatibility complex (MHC) region on chromosome 6. They all belong to MHC, which is related to many human traits. Obviously, these loci breached the polygenic assumption underlying. After deleting these loci, we reestimated the two kinds of heritability, and all the traits were basically close to the solid gray line and were closer compared with Figure 3 (see Supplementary Figure 4). This shows that the model assumptions were basically valid, and the estimated value of heritability had a certain degree of reliability.

Alternatively, the chromosome-wise partitioning heritability could be estimated jointly by fitting 22 autosomes altogether. It was basically the same as those calculated singly but slightly lower than the latter. It was because when calculating the heritability of chromosomes jointly, we set *N* for the whole genome in Equation (5), but smaller *N* were taken in the equation for estimating the heritability of each chromosome singly, as fewer individuals met the quality control standards for a single chromosome. We mentioned in Method that the fast estimation of joint heritability should meet the precondition that *N* of each chromosome are equal. The heritability estimated by the two methods will be strictly equal if this precondition holds (see Supplementary Notes). For traits with large sample sizes, this precondition could be met well, and the heritability estimated by the two methods was almost the same.

We also estimated the weighted chromosome-wise partition heritability and the weighted whole-genome heritability for these traits (see Supplementary Figure 5). In general, the weighted estimation of heritability was similar to that without weight.

## Discussion

In this study, we corrected the erroneous variance of the *L*_*B*_ estimator and proposed another two unbiased estimators of *tr*(***K***^*T*^***K***), which was the most time-consuming term in RHE (Wu and Sankararaman, 2018). Instead of plotting the running time and accuracy of different methods like most articles, we used a different experimental design to make a special comparison with the *L*_*B*_ estimator. We borrowed the sampling size parameter *B* in *L*_*B*_ and adjusted the sample size of our estimators so that the theoretical calculation time of the three estimators was the same under different sample size parameter *B*. Under the same time complexity, our results showed better stability with smaller variances. In other words, under the same accuracy requirements, our method could greatly reduce the computation cost.

We noted that Wu and Sankararaman further reduced the calculation time in matrix multiplication by introducing the mailman algorithm (Liberty and Zucker, 2009), which could also be used in our calculation by writing our estimators in the form of multiplication of genetic matrix and a random vector with multinoulli distribution. From these perspectives, our estimators were superior substitutions of the *L*_*B*_ estimator in Haseman–Elston regression.

We also gave the sampling variance of the subsampling estimator, which could be calculated by one sampling without additional calculation. As a result, the variance estimator of the heritability could be easily derived. Although the variance of the *L*_*B*_ estimator 2*tr* (***K***^*T*^***KK***^*T*^***K***)/*B* could also be derived by the subsampling method (beyond the scope of this study), its time complexity greatly exceeded the calculation of *tr* (***K***^*T*^***K***) as far as we know.

The variance of *L*_*S*_ was always slightly larger than that of *L*_*T*_. This was because *L*_*T*_ randomly extracted nearly uncorrelated elements in the lower triangular matrix ***K***_*o*_, while *L*_*S*_ extracted all elements in a triangle of ***K***_*o*_ (after reordering the individuals). Although their sampling variance was approximately equal to the population variance *var*(***K***_*o*_), the sampling variance of *L*_*T*_ was relatively smaller because it uses less related individuals.

One possible drawback of the *L*_*T*_ estimator relies on a much larger reference population than that of *L*_*S*_. When the reference sample size is small, it is obvious that *L*_*T*_ becomes *L*_*S*_. Therefore, the *L*_*T*_ estimator can make full use of large sample size, such as that of UKB. Although the difference between the variances of these two estimators is small, and the difference in the final heritability estimation is even slight, we still provide a novel and simple subsampling idea, which can be used in many situations involving large samples.

In the early analysis of heritability, both GRM and Haseman–Elston regression were applied to related individuals under the context of linkage analysis using sibling data. Under linkage, relatedness is actually related to the concept of identity by descent (IBD). However, with the increasing amount of data, the significance and application range of GRM and HE have been expanded. The unrelated individuals we emphasize here are mainly to distinguish from the linkage analysis of pedigree data. There is no problem in the estimation of heritability with related individuals, as demonstrated below. The expression $tr\left(K^{T}K\right)=N^{2}/M_{e}+N$ still holds true for related samples (see Supplementary Notes), which was confirmed in simulation (Supplementary Table 3). We have expanded the sample to all UKB British Whites, which included extra 131,850 individuals, totaling sample size *N* = 410,638, of various possible relatedness with the 278,788 unrelated samples and reestimated the heritability. The results are listed in Supplementary Table 4. In general, the heritability increased compared to the previous results of the unrelated set, but negligible. It shows that our estimators are basically applicable among a more realistic population even containing partially related individuals but leave some concerns in theoretical soundness.

Using modified Haseman–Elston regression to estimate heritability is becoming more and more popular in summary statistics. We further explored an important connection between Haseman–Elston regression and *M*_*e*_, the effective number of independent SNPs, which is also a critical concept in quantitative genetics. We found that *M*_*e*_ plays a pivotal role in the estimation of variance components and heritability. As long as we get the estimation of *M*_*e*_, we can easily get the estimation of its corresponding variance components.

Although we used only individual-level data to estimate heritability in this report, the nature of *M*_*e*_ allows researchers to estimate heritability based on a reference population of the same origin to the population in meta-analysis. However, the existence of family structure will make *M*_*e*_ shrink (see Supplementary Table 3; the expansion of trace means the shrinkage of *M*_*e*_), and different family structures make it shrink differently, leading to inaccurate meta-analysis. Therefore, we do not recommend using our method in samples with various related individuals, but it raises a very interesting question for the estimation theory using mega-scale family trees (Kaplanis et al., 2018; Shor et al., 2019).

Due to the statistical property of *M*_*e*_, we can easily extend *M*_*e*_ to the dominant model and use the same method to obtain both additive and dominant heritability, as long as their codes for the count of the reference allele are orthogonal, as discussed (Vitezica et al., 2017; Álvarez-Castro and Crujeiras, 2019). We can also extend *M*_*e*_ to estimate a genetic correlation for a pair of traits, in which $tr\left(K^{T}K\right)=N_{1}N_{2}/{\hat{M}}_{e}+N_{o}$, where *N*_*o*_ is the overlap sample size between a pair of cohorts, which have *N*_1_ and *N*_2_ individuals, respectively.

**URLs**: The related source code, https://github.com/GuoanQi1996/LT-Estimator.

## Data Availability Statement

Publicly available datasets were analyzed in this study. This data can be found at: https://biobank.ctsu.ox.ac.uk/.

## Ethics Statement

Ethical review and approval was not required for the study on human participants in accordance with the local legislation and institutional requirements. The patients/participants provided their written informed consent to participate in this study.

## Author Contributions

TX developed the method and wrote the manuscript. G-AQ analyzed the data. JZ and H-MX revised the manuscript. G-BC designed the study and improved the manuscript. All authors read and approved the final manuscript.

## Conflict of Interest

The authors declare that the research was conducted in the absence of any commercial or financial relationships that could be construed as a potential conflict of interest.
