# Supplementary material for: Subsampling Technique to Estimate Variance Component for UK-Biobank Traits
Source: Front Genet. 2021 Mar 5;12:612045. doi: 10.3389/fgene.2021.612045 (PMC7978110; doi:10.3389/fgene.2021.612045)
Supplement: Supplementary file 1 [file Data_Sheet_1.pdf]

Supplementary Notes and Figures & Tables

Table of Contents

Supplementary Notes **2**

S1. The derivation of $\mathrm{tr}(K^T K) = N + N^2/M_e$ 2

S2. The expectation of the L_S estimator 2

S3. The expectation and sampling variance of the L_T estimator 2

S4. Method-of-Moments 2

S5. Estimating chromosome-wise partition heritability jointly 4

Supplementary Figures **5**

Figure S1. The sampling variances of three estimators under different parameter settings with weak LD. 5

Figure S2. The sampling variances of three estimators under different parameter settings with strong LD. 7

Figure S3. The means and the sampling variances of three estimators under real-data simulation. 9

Figure S4. The relationship of the chromosome-wise partition heritability and the whole-genome heritability after deleting SNPs related to MHC. 10

Figure S5. The relationship of the weighted chromosome-wise partition heritability and the weighted whole-genome heritability. 11

Supplementary Tables **13**

Table S1. The actual computational time (minutes) of the real data calculation. 13

Table S2. Estimation heritability of 81 traits in the UKB dataset. 14

Table S3. The means and sampling variance of the three estimators 16

Table S4. Estimation heritability of 81 traits in the UKB dataset for unrelated and related individuals. 17

Supplementary Notes and Figures & Tables

Supplementary Notes

S1. The derivation of $tr(\boldsymbol{K}^T\boldsymbol{K}) = \boldsymbol{N} + \boldsymbol{N}^2/\boldsymbol{M}_e$

$$tr(\boldsymbol{K}^T\boldsymbol{K}) = \sum\nolimits_{i,j}^{N} \boldsymbol{K}_{i,j}^2 = N + 2\sum_{i=1}^{N-1}\sum_{j>i}^{N} \boldsymbol{K}_{i,j}^2 = N + 2\sum_{i=1}^{N-1}\sum_{j>i}^{N} (\boldsymbol{K}_{i,j} - \overline{\boldsymbol{K}_o})^2 = N + N^2/M_e$$

where $\overline{\boldsymbol{K}_o}$ is the mean value of $\boldsymbol{K}_o$. Under the premise of standardization of the genotype matrix, the sum of all elements of $\boldsymbol{K}$ should be 0, and $\mathbb{E}\big(tr(\boldsymbol{K})\big) = N - 1$ for any samples (unrelated or with related). So $\overline{\boldsymbol{K}_o}$ is always equal to 0 ($-1/N$ actually, and approximate to 0), and the last step holds true.

S2. The expectation of the $\boldsymbol{L_S}$ estimator

$$\mathbb{E}(L_S) = \mathbb{E}\big(N^2\widehat{1/M_e} + N\big) = N + N^2\mathbb{E}\big(Var(\boldsymbol{K}_s)\big) = N + N^2 Var(\boldsymbol{K}_o) = N + N^2/M_e$$

$$= N + 2\sum_{i=1}^{N-1}\sum_{j>i}^{N} (\boldsymbol{K}_{i,j} - \overline{\boldsymbol{K}_o})^2 = N + 2\sum_{i=1}^{N-1}\sum_{j>i}^{N} \boldsymbol{K}_{i,j}^2 = \sum\nolimits_{i,j}^{N} \boldsymbol{K}_{i,j}^2 = tr(\boldsymbol{K}^T\boldsymbol{K})$$

where $\boldsymbol{K}_s$ is the off-diagonal elements of GRM matrix of sampling individuals.

S3. The expectation and sampling variance of the $\mathbf{L_T}$ estimator

$$\mathbb{E}(L_T) = \mathbb{E}\left(N + \frac{N^2}{n}\sum\nolimits_{i=1}^{n} {\boldsymbol{K}_o}_{A_i}^2\right) = N + \frac{N^2}{n}\mathbb{E}\left(\sum\nolimits_{i=1}^{n} {\boldsymbol{K}_o}_{A_i}^2\right) = N + N^2\mathbb{E}\left(\sum\nolimits_{i=1}^{N(N-1)/2} {\boldsymbol{K}_o}_i^2\right)$$

$$= N + 2\sum_{i=1}^{N-1}\sum_{j>i}^{N} \boldsymbol{K}_{i,j}^2 = \sum\nolimits_{i,j}^{N} \boldsymbol{K}_{i,j}^2 = tr(\boldsymbol{K}^T\boldsymbol{K})$$

$$var(L_T) = var\left(N + \frac{N^2}{n}\sum\nolimits_{i=1}^{n} {\boldsymbol{K}_o}_{A_i}^2\right) = \frac{N^4}{n^2} var\left(\sum\nolimits_{i=1}^{n} {\boldsymbol{K}_o}_{A_i}^2\right) = N^4 var({\boldsymbol{K}_o}^2)/n$$

S4. Method-of-Moments

We assume that

$$\boldsymbol{y} = \boldsymbol{X\beta} + \epsilon, \boldsymbol{\beta} \sim \mathcal{N}\left(0, \frac{h^2}{M}\boldsymbol{I}_M\right), \epsilon \sim \mathcal{N}(0, \sigma_e^2\boldsymbol{I}_N)$$

where $\boldsymbol{y}$ is the standard phenotype vector, $\boldsymbol{X}$ is the standard genotypic matrix with M markers and N individuals. h^2 is the genetic variance component and σ_e^2 is the residual variance. The MoM (Method-of-Moments) estimator of HE-regression is calculated from the equations:

$$\begin{bmatrix} tr(\boldsymbol{K}^T\boldsymbol{K}) & tr(\boldsymbol{K}) \\ tr(\boldsymbol{K}) & N \end{bmatrix} \begin{bmatrix} \widehat{h^2} \\ \widehat{\sigma_e^2} \end{bmatrix} = \begin{bmatrix} \boldsymbol{y}^T\boldsymbol{K}\boldsymbol{y} \\ \boldsymbol{y}^T\boldsymbol{y} \end{bmatrix}.$$

the estimator for variance component σ_g^2 can be written as

$$\widehat{h^2} = \frac{\boldsymbol{y}^T(\boldsymbol{K}-\boldsymbol{I})\boldsymbol{y}}{tr(\boldsymbol{K}^T\boldsymbol{K}) - N}$$

Define $\widetilde{h^2}$ the plug-in estimator of h^2 by estimating $tr(\boldsymbol{K}^T\boldsymbol{K})$ with $tr\widehat{(\boldsymbol{K}^T\boldsymbol{K})}$ via L_T estimator. We have,

$$\mathbb{E}(\boldsymbol{y}^T(\boldsymbol{K}-\boldsymbol{I})\boldsymbol{y}) = \mathbb{E}(\boldsymbol{y}^T\boldsymbol{K}\boldsymbol{y} - \boldsymbol{y}^T\boldsymbol{y}) = \mathbb{E}(tr(\boldsymbol{y}^T\boldsymbol{K}\boldsymbol{y}) - tr(\boldsymbol{y}^T\boldsymbol{y})) = tr(\mathbb{E}(\boldsymbol{y}\boldsymbol{y}^T)\boldsymbol{K}) - tr\big(\mathbb{E}(\boldsymbol{y}\boldsymbol{y}^T)\big)$$
$$= tr\big((h^2\boldsymbol{K} + \sigma_e^2\boldsymbol{I})\boldsymbol{K} - (h^2\boldsymbol{K} + \sigma_e^2\boldsymbol{I})\big) = tr(h^2\boldsymbol{K}\boldsymbol{K} + \sigma_e^2\boldsymbol{K} - h^2\boldsymbol{K} - \sigma_e^2\boldsymbol{I})$$
$$= h^2 tr(\boldsymbol{K}^T\boldsymbol{K}) + N\sigma_e^2 - Nh^2 - N\sigma_e^2 = h^2(tr(\boldsymbol{K}^T\boldsymbol{K}) - N)$$

For a variable X with expectation μ and variance σ^2, the expectation of $1/X$ can be approximated as $(1+\sigma^2/\mu^2)/\mu$. Using this property, we can get

$$\mathbb{E}\big(\widetilde{h^2}\big) = h^2(tr(\boldsymbol{K}^T\boldsymbol{K}) - N)\mathbb{E}\left(\frac{1}{tr\widehat{(\boldsymbol{K}^T\boldsymbol{K})} - N}\right) = h^2\left(1 + \frac{var\big(tr\widehat{(\boldsymbol{K}^T\boldsymbol{K})} - N\big)}{\mathbb{E}\big(tr\widehat{(\boldsymbol{K}^T\boldsymbol{K})} - N\big)^2}\right)$$
$$= h^2\left(1 + \frac{N^4 var({\boldsymbol{K}_o}^2)/n}{\mathbb{E}\big(tr\widehat{(\boldsymbol{K}^T\boldsymbol{K})} - N\big)^2}\right) = h^2 + \frac{1}{n}\frac{N^4 var({\boldsymbol{K}_o}^2)}{(tr(\boldsymbol{K}^T\boldsymbol{K}) - N)^2}h^2 = h^2 + \frac{var({\boldsymbol{K}_o}^2){M_e}^2}{n}h^2$$

For two independent variable X and Y with expectation μ_x, μ_y and variance σ_x^2, σ_y^2, the variance of X/Y can be approximated as $\sigma_x^2/\mu_y^2 + \mu_x^2\sigma_y^2/\mu_y^4$. We also have,

$$var(\boldsymbol{y}^T(\boldsymbol{K}-\boldsymbol{I})\boldsymbol{y}) = 2tr\big(\boldsymbol{\Sigma}(\boldsymbol{K}-\boldsymbol{I})\boldsymbol{\Sigma}(\boldsymbol{K}-\boldsymbol{I})\big) = 2tr\big((h^2\boldsymbol{K} + \sigma_e^2\boldsymbol{I})(\boldsymbol{K}-\boldsymbol{I})(h^2\boldsymbol{K} + \sigma_e^2\boldsymbol{I})(\boldsymbol{K}-\boldsymbol{I})\big)$$
$$\approx 2tr\big(\boldsymbol{I}(\boldsymbol{K}-\boldsymbol{I})\boldsymbol{I}(\boldsymbol{K}-\boldsymbol{I})\big) = \frac{2N^2}{M_e}$$

Using this property, we can get

$$var\big(\widetilde{h^2}\big) = \frac{var(\boldsymbol{y}^T(\boldsymbol{K}-\boldsymbol{I})\boldsymbol{y})}{(tr(\boldsymbol{K}^T\boldsymbol{K}) - N)^2} + \frac{\mathbb{E}(\boldsymbol{y}^T(\boldsymbol{K}-\boldsymbol{I})\boldsymbol{y})^2 N^4 var({\boldsymbol{K}_o}^2)/n}{(tr(\boldsymbol{K}^T\boldsymbol{K}) - N)^4} \approx \frac{2M_e}{N^2} + \frac{var\big({\boldsymbol{K}_o}^2\big){M_e}^2}{n}(h^2)^2$$

and

$$\widehat{var\big(\widetilde{h^2}\big)} = \frac{2\widehat{M_e}}{N^2} + \frac{var\left({\boldsymbol{K}_{o}}_{A_i}^2\right)\widehat{M_e}^2}{n}(\widetilde{h^2})^2$$

S5. Estimating chromosome-wise partition heritability jointly

The moment estimator is to minimize

$$\boldsymbol{Q} = tr\{[\boldsymbol{y}\boldsymbol{y}^T - (h^2\boldsymbol{K} + \sigma_e^2\boldsymbol{I})]^2\}$$

We can expand the term $h^2\boldsymbol{K}$ into $h_1^2\boldsymbol{K}_1 + h_2^2\boldsymbol{K}_2 + \cdots + h_{22}^2\boldsymbol{K}_{22}$, in which $\boldsymbol{K}_c$ is the $N \times N$ GRM for the cth chromosome. By taking the differentiation in terms of h_c^2 and σ_e^2, we have

$$\begin{cases} \dfrac{\partial \boldsymbol{Q}}{\partial h_c^2} = tr\{h_1^2\boldsymbol{K}_1^T\boldsymbol{K}_c + h_2^2\boldsymbol{K}_2^T\boldsymbol{K}_c + \cdots + h_{22}^2\boldsymbol{K}_{22}^T\boldsymbol{K}_c + \sigma_e^2\boldsymbol{K}_c - \boldsymbol{y}\boldsymbol{y}^T\boldsymbol{K}_c\} = 0 \\ \dfrac{\partial \boldsymbol{Q}}{\partial \sigma_e^2} = tr\{h_1^2\boldsymbol{K}_1 + h_2^2\boldsymbol{K}_2 + \cdots + h_{22}^2\boldsymbol{K}_{22} + \sigma_e^2\boldsymbol{I} - \boldsymbol{y}\boldsymbol{y}^T\boldsymbol{I}\} = 0 \end{cases}$$

We can organize it into the matrix form

$$\begin{bmatrix} (tr(\boldsymbol{K}_i^T\boldsymbol{K}_j)) & N\boldsymbol{1} \\ N\boldsymbol{1}^T & N \end{bmatrix} \begin{bmatrix} \widehat{\boldsymbol{h}}_c^2 \\ \hat{\sigma}_e^2 \end{bmatrix} = \begin{bmatrix} (\boldsymbol{y}^T\boldsymbol{K}_c\boldsymbol{y}) \\ \boldsymbol{y}^T\boldsymbol{I}\boldsymbol{y} \end{bmatrix}$$

where $(tr(\boldsymbol{K}_i^T\boldsymbol{K}_j))$ is a 22×22 matrix with each element $tr(\boldsymbol{K}_i^T\boldsymbol{K}_j)$, $\boldsymbol{1}$ is the vector with all ones, $\widehat{\boldsymbol{h}}_c^2$ is the vector for joint chromosome-wise partition heritability, and $(\boldsymbol{y}^T\boldsymbol{K}_c\boldsymbol{y})$ is a vactor with each element $\boldsymbol{y}^T\boldsymbol{K}_c\boldsymbol{y}$.

Easy to prove that $tr(\boldsymbol{K}_i^T\boldsymbol{K}_j) = N$ if $i \neq j$, and $tr(\boldsymbol{K}_c^T\boldsymbol{K}_c) \triangleq N^2/M_{e.c} + N$. This equation is easy to solve, because the inverse of the matrix on the left side of the above formula can be easily obtained

$$\begin{bmatrix} (tr(\boldsymbol{K}_i^T\boldsymbol{K}_j)) & N\boldsymbol{1} \\ N\boldsymbol{1}^T & N \end{bmatrix}^{-1} = \begin{bmatrix} M_{e.1}/N^2 & 0 & \cdots & 0 & -M_{e.1}/N^2 \\ 0 & M_{e.2}/N^2 & \cdots & 0 & -M_{e.2}/N^2 \\ \vdots & \vdots & \ddots & \vdots & \vdots \\ 0 & 0 & \cdots & M_{e.22}/N^2 & -M_{e.22}/N^2 \\ -M_{e.1}/N^2 & -M_{e.2}/N^2 & \cdots & -M_{e.22}/N^2 & (N + \sum M_{e.c})/N^2 \end{bmatrix}$$

Supplementary Notes and Figures & Tables

Supplementary Figures

Figure S1. The sampling variances of three estimators under different parameter settings with weak LD.

The genotype data were constituted by N individuals and M markers, and the specific value of them was written in the title of each small figure. B is the parameter related to the sample size in the L_B estimator. We set different B, and for each B the number of samples of the L_S and L_T estimators were adjusted to ensure the three estimators are calculated for an equal computational cost. The red dots represent the variances of the L_B estimator; the green dots represent the variances of the L_S estimator; the blue dots represent the variances of the L_T estimator. The solid red line represents the theoretical sampling variance of the L_B estimator; the longdash red line represents the error sampling variance of the L_B estimator given by Wu and Sankararaman; the blue line represent the theoretical sampling variance of the L_T estimator.

Supplementary Notes and Figures & Tables

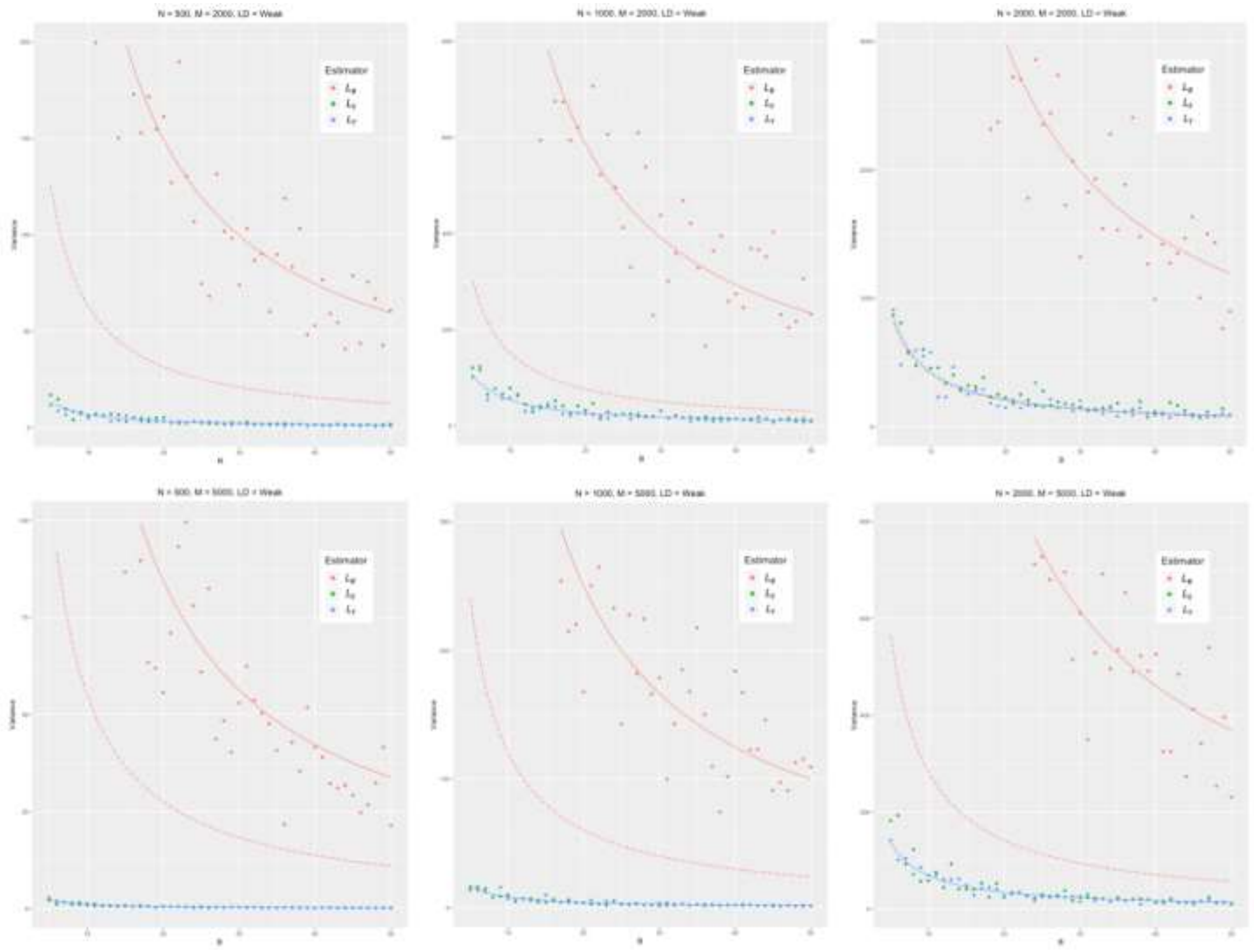

Figure S2. The sampling variances of three estimators under different parameter settings with strong LD.

The genotype data were constituted by N individuals and M markers, and the specific value of them was written in the title of each small figure. B is the parameter related to the sample size in the L_B estimator. We set different B, and for each B the number of samples of the L_S and L_T estimators were adjusted to ensure the three estimators are calculated for an equal computational cost. The red dots represent the variances of the L_B estimator; the green dots represent the variances of the L_S estimator; the blue dots represent the variances of the L_T estimator. The solid red line represents the theoretical sampling variance of the L_B estimator; the longdash red line represents the error sampling variance of the L_B estimator given by Wu and Sankararaman; the blue line represent the theoretical sampling variance of the L_T estimator.

Supplementary Notes and Figures & Tables

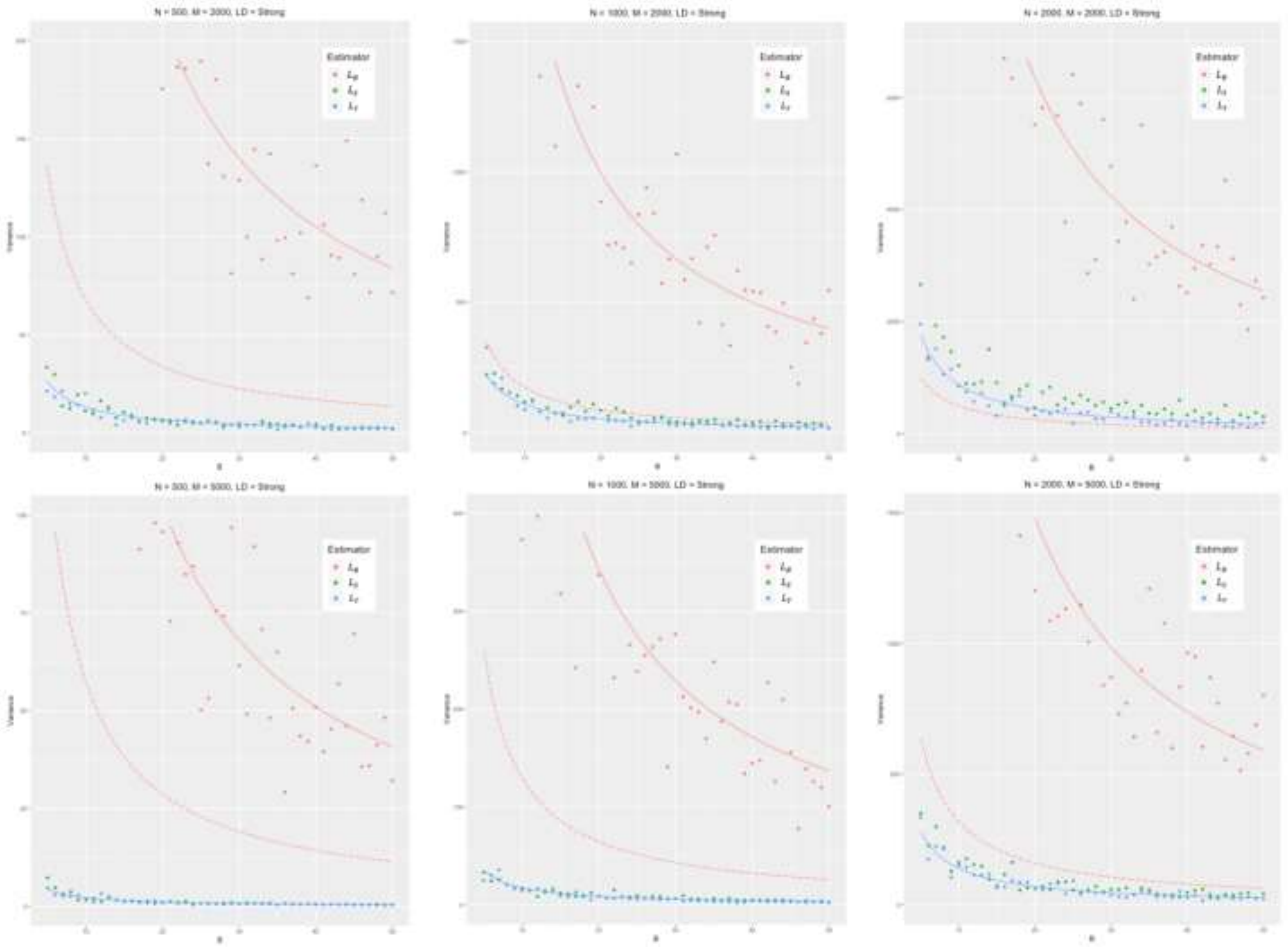

Figure S3. The means and the sampling variances of three estimators under real-data simulation. The genotype data consists of 12,980 adjacent markers on chromosome 22 of 2,000 individuals randomly sampled form the UK Biobank data. B is the parameter related to the sample size in the L_B estimator, which was set from 5 to 50, and repeated 100 times for each to assess the mean and variance of the three estimators. (**A**) The means of 100 random experiments of three extimators for each B. Red dots represent the means of the L_B estimator; green dots represent the means of the L_S estimator; blue dots represent the means of the L_T estimator. The black line represents the real $tr(\boldsymbol{K}^2)$. (**B**) The log_2-variances of 100 random experiments of three extimators for each B. Since the variances of three estimators differ too much, we have performed log conversion on them for the convenience of display. The red dots represent the log_2- variances of the L_B estimator; the green dots represent the log_2- variances of the L_S estimator; the blue dots represent the log_2- variances of the L_T estimator. The solid red line represents the theoretical sampling log_2- variance of the L_B estimator; the longdash red line represents the error sampling log_2- variance of the L_B estimator given by Wu; the blue line represent the theoretical sampling log_2- variance of the L_T estimator.

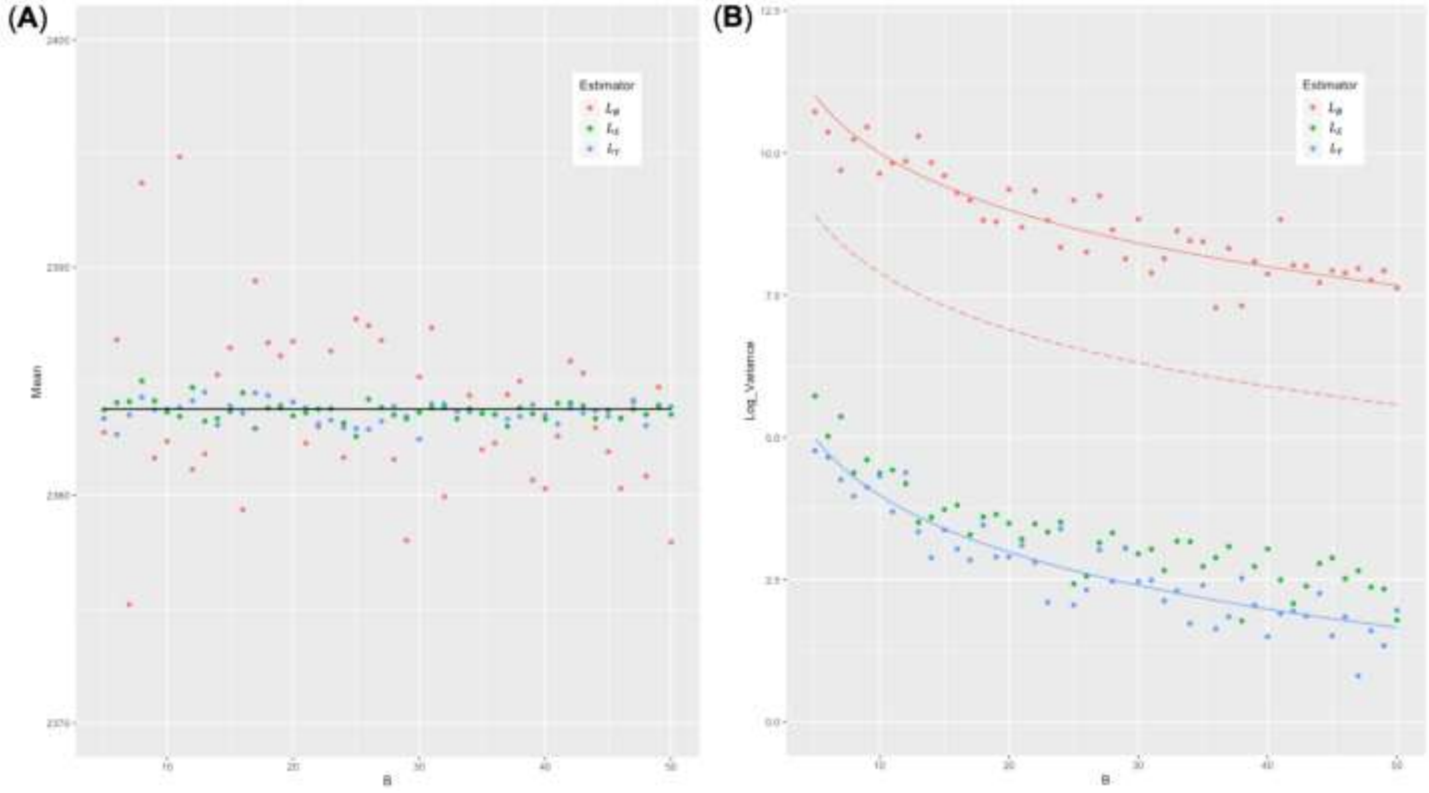

Figure S4. The relationship of the chromosome-wise partition heritability and the whole-genome heritability after deleting SNPs related to MHC.

Each dot represents a trait in the UKB dataset listed in Table S2. The horizontal axis represents their weighted chromosome-wise partition heritability, and the vertical line at each point is their error bar; the vertical axis represents their weighted whole-genome heritability. The long-dash line crosses the origin has a slope of 1. The slop of the solid line is 0.837, the ratio of $\frac{\widehat{M}_e}{\sum_{c=1}^{22}\widehat{m}_{e.c}}$.

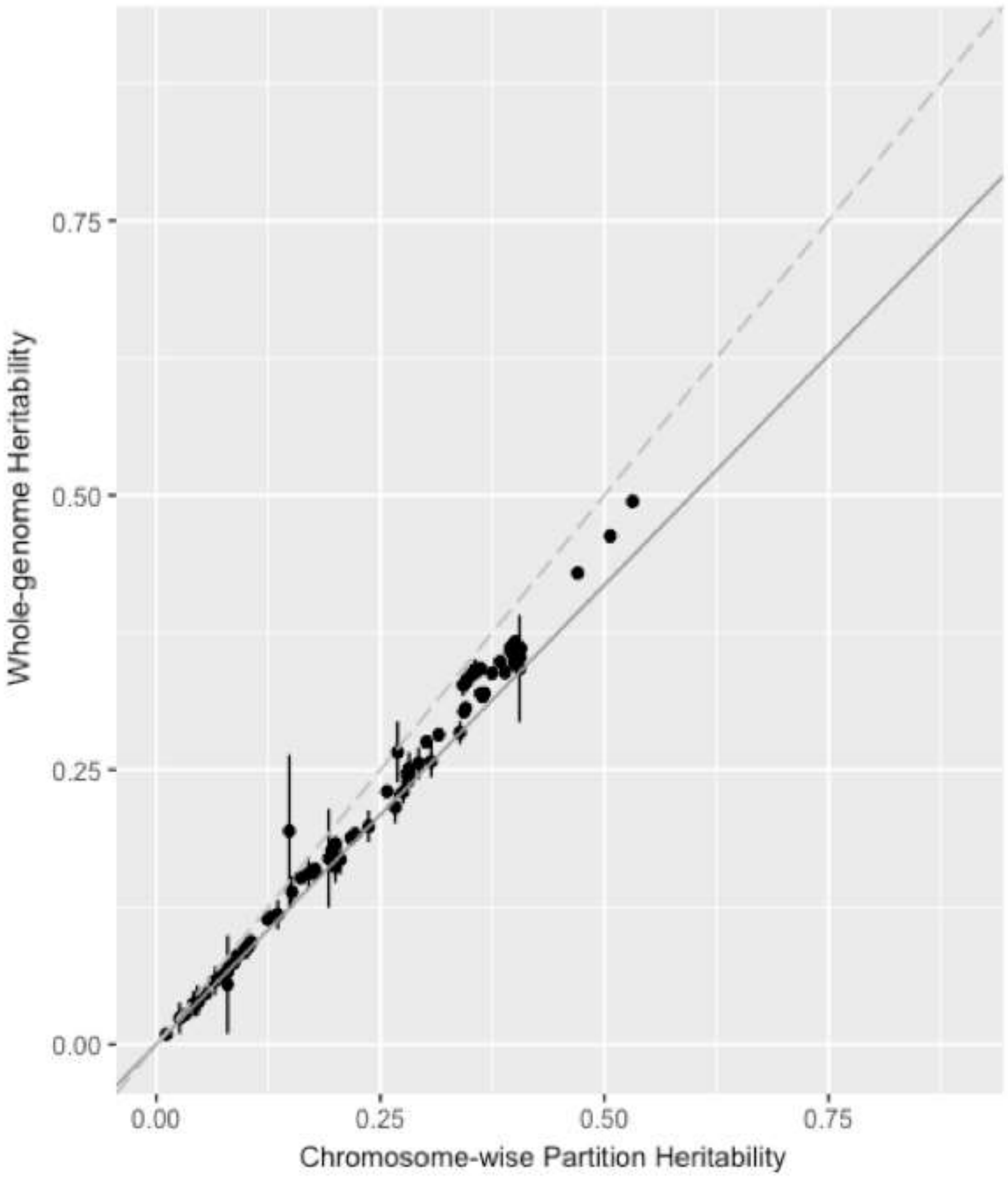

Figure S5. The relationship of the weighted chromosome-wise partition heritability and the weighted whole-genome heritability.

Each dot represents a trait in the UKB dataset listed in Table S2. The horizontal axis represents their weighted chromosome-wise partition heritability, and the vertical line at each point is their error bar; the vertical axis represents their weighted whole-genome heritability. The red colour represents the weighted chromosome-wise partition heritability calculated jointly, and the green colour represents the weighted chromosome-wise partition heritability calculated singly. The long-dash line crosses the origin has a slope of 1. The slop of the solid line is 0.644, the ratio of $\frac{\hat{M}_{ew}}{\sum_{c=1}^{22}\hat{m}_{ew.c}}$.

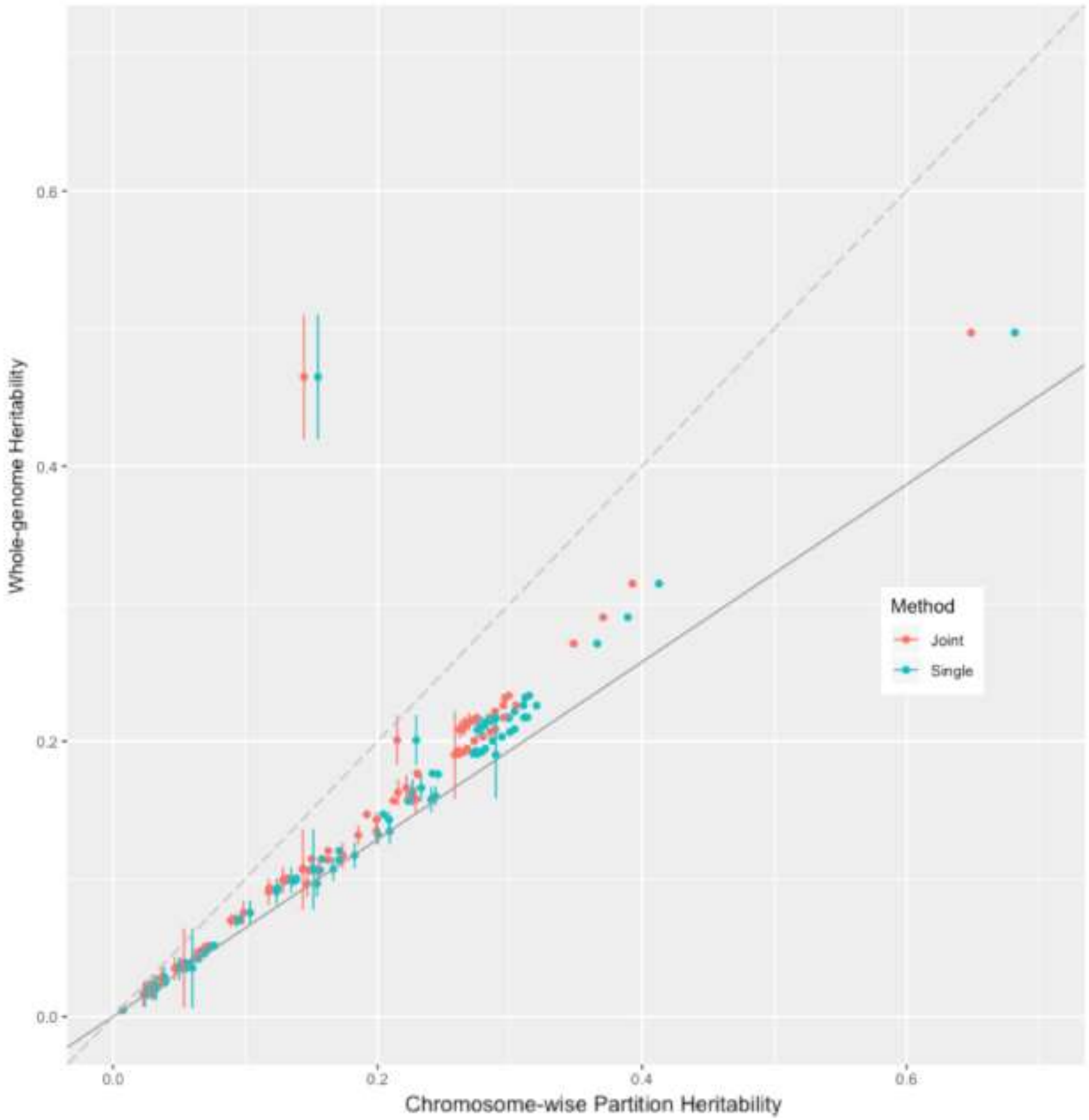
Whole-genome Heritability
0.0
0.2
0.4
0.6
Chromosome-wise Partition Heritability
Method
Joint
Single

Supplementary Notes and Figures & Tables

Supplementary Tables

Table S1. The actual computational time (minutes) of the real data calculation.

B represents the iterations taken by L_B. We took sample size $s = \sqrt{2BN}$ for L_S and $n = BN$ for L_T in each step to guarantee the three estimators having the equal computational cost of $\mathcal{O}(NMB)$, where N is the total sample size.

B	L_B	L_S	L_T
5	80	60	60
10	170	100	110
20	330	210	220
40	640	440	470
60	930	630	670
80	1,220	840	890
100	1,540	1,050	1,110

The computational time of our two estimators are similar, and L_T takes a little longer mainly due to the extra cycle for picking random samples; L_B takes half more time than ours, and we guess the possible reason is that although the theoretical calculation complexity is $\mathcal{O}(NMB)$, the vector and matrix are multiplied twice actually (see **Sampling method I**). Excluding this influence, the actual calculation time of the three estimators are consistent with the theoretical calculation complexity.

Table S2. Estimation heritability of 81 traits in the UKB dataset.

N is the sample size, $\hat{h}^2_{Chr}$ is the chromosome-wise partition heritability calculated by adding the heritability of each chromosome, $\hat{h}^2_{Gen}$ is the whole-genome heritability calculated from the GRM of the whole genome, and $\hat{h}^2_{se}$ is the standard error of the whole-genome heritability. Above the dotted line are physiological traits, and social traits are below the dotted line.

Field ID	Field Name	Category	N	$\hat{h}^2_{Chr}$	$\hat{h}^2_{Gen}$	$\hat{h}^2_{se}$
21021	Pulse wave Arterial Stiffness index	Arterial stiffness	92,137	0.045	0.033	0.005
102	Pulse rate, automated reading	Blood pressure	259,815	0.217	0.170	0.002
4079	Diastolic blood pressure, automated reading	Blood pressure	259,815	0.199	0.161	0.002
4080	Systolic blood pressure, automated reading	Blood pressure	259,812	0.194	0.155	0.002
48	Waist circumference	Body size measures	277,649	0.278	0.219	0.002
49	Hip circumference	Body size measures	277,613	0.300	0.244	0.002
50	Standing height	Body size measures	277,508	0.895	0.729	0.002
20015	Sitting height	Body size measures	277,231	0.528	0.444	0.002
21001	Body mass index (BMI)	Body size measures	277,223	0.360	0.282	0.002
21002	Weight	Body size measures	277,325	0.372	0.299	0.002
78	Heel bone mineral density (BMD) T-score, automated	Bone-densitometry of heel	158,445	0.355	0.299	0.003
3148	Heel bone mineral density (BMD)	Bone-densitometry of heel	158,371	0.359	0.301	0.003
4105	Heel bone mineral density (BMD) (left)	Bone-densitometry of heel	89,026	0.343	0.288	0.005
4106	Heel bone mineral density (BMD) T-score, automated (left)	Bone-densitometry of heel	89,076	0.339	0.286	0.005
4124	Heel bone mineral density (BMD) (right)	Bone-densitometry of heel	89,025	0.352	0.296	0.005
4125	Heel bone mineral density (BMD) T-score, automated (right)	Bone-densitometry of heel	89,071	0.348	0.293	0.005
20022	Birth weight	Early life factors	159,328	0.176	0.141	0.003
46	Hand grip strength (left)	Hand grip strength	277,019	0.178	0.142	0.002
47	Hand grip strength (right)	Hand grip strength	277,027	0.176	0.143	0.002
20019	Speech-reception-threshold (SRT) estimate (left)	Hearing test	84,777	0.042	0.033	0.005
20021	Speech-reception-threshold (SRT) estimate (right)	Hearing test	84,880	0.047	0.034	0.005
23099	Body fat percentage	Impedance measures	273,077	0.387	0.298	0.002
23102	Whole body water mass	Impedance measures	273,248	0.468	0.386	0.002
23106	Impedance of whole body	Impedance measures	273,221	0.406	0.321	0.002
23107	Impedance of leg (right)	Impedance measures	273,237	0.404	0.314	0.002
23108	Impedance of leg (left)	Impedance measures	273,233	0.399	0.313	0.002
23109	Impedance of arm (right)	Impedance measures	273,218	0.343	0.269	0.002
23110	Impedance of arm (left)	Impedance measures	273,232	0.344	0.272	0.002
23111	Leg fat percentage (right)	Impedance measures	273,234	0.404	0.311	0.002
23114	Leg predicted mass (right)	Impedance measures	273,224	0.393	0.319	0.002
23115	Leg fat percentage (left)	Impedance measures	273,218	0.397	0.306	0.002
23118	Leg predicted mass (left)	Impedance measures	273,202	0.382	0.310	0.002
23119	Arm fat percentage (right)	Impedance measures	273,196	0.362	0.279	0.002
23122	Arm predicted mass (right)	Impedance measures	273,167	0.399	0.329	0.002
23123	Arm fat percentage (left)	Impedance measures	273,155	0.364	0.281	0.002
23126	Arm predicted mass (left)	Impedance measures	273,112	0.394	0.326	0.002
23127	Trunk fat percentage	Impedance measures	273,083	0.362	0.278	0.002
23130	Trunk predicted mass	Impedance measures	272,987	0.505	0.418	0.002
5254	Intra-ocular pressure, corneal-compensated (right)	Intraocular pressure	60,140	0.151	0.125	0.007
5255	Intra-ocular pressure, Goldmann-correlated (right)	Intraocular pressure	60,140	0.282	0.227	0.007
5256	Corneal hysteresis (right)	Intraocular pressure	60,140	0.236	0.173	0.007
5257	Corneal resistance factor (right)	Intraocular pressure	60,140	0.307	0.228	0.007
5262	Intra-ocular pressure, corneal-compensated (left)	Intraocular pressure	60,048	0.169	0.139	0.007
5263	Intra-ocular pressure, Goldmann-correlated (left)	Intraocular pressure	60,048	0.293	0.233	0.007
5264	Corneal hysteresis (left)	Intraocular pressure	60,048	0.199	0.139	0.007
5265	Corneal resistance factor (left)	Intraocular pressure	60,048	0.267	0.192	0.007

2966	Age high blood pressure diagnosed	Medical conditions	73,517	0.042	0.035	0.006
2976	Age diabetes diagnosed	Medical conditions	12,628	0.231	0.618	0.033
3761	Age hay fever, rhinitis or eczema diagnosed	Medical conditions	64,854	0.136	0.111	0.006
3786	Age asthma diagnosed	Medical conditions	31,535	0.271	0.265	0.013
20127	Neuroticism score	Mental health	226,198	0.160	0.133	0.002
20150	Forced expiratory volume in 1-second (FEV1), Best measure	Spirometry	207,848	0.257	0.207	0.002
20151	Forced vital capacity (FVC), Best measure	Spirometry	207,848	0.314	0.252	0.002
5201	logMAR, final (right)	Visual acuity	62,296	0.026	0.021	0.007
5208	logMAR, final (left)	Visual acuity	62,255	0.045	0.034	0.007
189	Townsend deprivation index at recruitment	Baseline characteristics	277,798	0.280	0.209	0.002
1289	Cooked vegetable intake	Diet	278,142	0.079	0.062	0.002
1299	Salad / raw vegetable intake	Diet	278,142	0.079	0.057	0.002
1309	Fresh fruit intake	Diet	278,142	0.074	0.056	0.002
1438	Bread intake	Diet	277,852	0.098	0.073	0.002
1488	Tea intake	Diet	278,142	0.103	0.079	0.002
1528	Water intake	Diet	278,142	0.098	0.075	0.002
2714	Age when periods started (menarche)	Female-specific factors	148,818	0.127	0.103	0.003
2744	Birth weight of first child	Female-specific factors	120,455	0.124	0.102	0.003
2754	Age at first live birth	Female-specific factors	100,951	0.281	0.217	0.004
3872	Age of primiparous women at birth of child	Female-specific factors	19,504	0.193	0.155	0.021
874	Duration of walks	Physical activity	267,826	0.055	0.040	0.002
894	Duration of moderate activity	Physical activity	231,311	0.045	0.034	0.002
914	Duration of vigorous activity	Physical activity	166,696	0.032	0.025	0.003
1070	Time spent watching television (TV)	Physical activity	278,142	0.221	0.167	0.002
2139	Age first had sexual intercourse	Sexual factors	255,880	0.064	0.051	0.002
2149	Lifetime number of sexual partners	Sexual factors	253,460	0.011	0.008	0.002
1160	Sleep duration	Sleep	278,142	0.089	0.070	0.002
2867	Age started smoking in former smokers	Smoking	66,988	0.065	0.049	0.006
2887	Number of cigarettes previously smoked daily	Smoking	63,560	0.205	0.149	0.007
3436	Age started smoking in current smokers	Smoking	19,655	0.081	0.049	0.021
3456	Number of cigarettes currently smoked daily (current cigarette smokers)	Smoking	18,005	0.404	0.294	0.023
20161	Pack years of smoking	Smoking	81,555	0.275	0.201	0.005
20162	Pack years adult smoking as proportion of life span exposed to smoking	Smoking	81,555	0.338	0.248	0.005
1050	Time spend outdoors in summer	Sun exposure	277,852	0.105	0.079	0.002
1060	Time spent outdoors in winter	Sun exposure	277,852	0.086	0.063	0.002

Table S3. The means and sampling variance of the three estimators.

We set $N = M = 2{,}000$ with 50% subjects unrelated with each other, and the other have one relative (500 sibpairs). The kinship coefficient was set equal to 0 (unrelated samples), 0.125, 0.25, and 0.5. We set $B = 50$, and took sample size $s = \sqrt{2BN}$ for L_S and $n = BN$ for L_T to guarantee the three estimators having the equal computational cost of $\mathcal{O}(NMB)$. The simulation repeated 100 times to assess the mean and variance of the three estimators. Trace represents the real value of $tr(\boldsymbol{K}^T\boldsymbol{K})$.

Kinship Coefficient	Estimator	L_B		L_S		L_T	
	Trace	Mean	Variance	Mean	Variance	Mean	Variance
0	4,064.26	4,065.92	834.64	4,061.32	98.83	4,065.23	90.19
0.125	4,133.40	4,137.08	945.29	4,133.71	494.04	4,135.07	106.07
0.25	4,359.24	4,366.53	1,188.19	4,364.22	2,489.73	4,358.20	654.68
0.5	5,175.74	5,177.63	3,364.10	5,180.77	10,863.06	5,173.93	17,893.06

Table S4. Estimation heritability of 81 traits in the UKB dataset for unrelated and related individuals.

Unrelated represents the estimation heritability for 278,788 unrelated samples, With related represents the estimation heritability for 410,638 samples with 278,788 unrelated individuals and 131,850 related individuals.

Field ID	Field Name	Unrelated	With related
46	Hand grip strength (left)	0.142	0.139
47	Hand grip strength (right)	0.143	0.140
48	Waist circumference	0.219	0.223
49	Hip circumference	0.244	0.250
50	Standing height	0.729	0.737
78	Heel bone mineral density (BMD) T-score, automated	0.299	0.295
102	Pulse rate, automated reading	0.170	0.169
189	Townsend deprivation index at recruitment	0.209	0.233
874	Duration of walks	0.040	0.040
894	Duration of moderate activity	0.034	0.036
914	Duration of vigorous activity	0.025	0.021
1050	Time spend outdoors in summer	0.079	0.080
1060	Time spent outdoors in winter	0.063	0.065
1070	Time spent watching television (TV)	0.167	0.167
1160	Sleep duration	0.070	0.072
1289	Cooked vegetable intake	0.062	0.064
1299	Salad / raw vegetable intake	0.057	0.056
1309	Fresh fruit intake	0.056	0.055
1438	Bread intake	0.073	0.073
1488	Tea intake	0.079	0.082
1528	Water intake	0.075	0.075
2139	Age first had sexual intercourse	0.051	0.051
2149	Lifetime number of sexual partners	0.008	0.005
2714	Age when periods started (menarche)	0.103	0.106
2744	Birth weight of first child	0.102	0.103
2754	Age at first live birth	0.217	0.217
2867	Age started smoking in former smokers	0.049	0.049
2887	Number of cigarettes previously smoked daily	0.149	0.156
2966	Age high blood pressure diagnosed	0.035	0.036
2976	Age diabetes diagnosed	0.618	0.612
3148	Heel bone mineral density (BMD)	0.301	0.298
3436	Age started smoking in current smokers	0.049	0.044
3456	Number of cigarettes currently smoked daily (current cigarette smokers)	0.294	0.279
3761	Age hay fever, rhinitis or eczema diagnosed	0.111	0.120
3786	Age asthma diagnosed	0.265	0.228
3872	Age of primiparous women at birth of child	0.155	0.168
4079	Diastolic blood pressure, automated reading	0.161	0.162
4080	Systolic blood pressure, automated reading	0.155	0.155
4105	Heel bone mineral density (BMD) (left)	0.288	0.293
4106	Heel bone mineral density (BMD) T-score, automated (left)	0.286	0.291
4124	Heel bone mineral density (BMD) (right)	0.296	0.311
4125	Heel bone mineral density (BMD) T-score, automated (right)	0.293	0.308
5201	logMAR, final (right)	0.021	0.025
5208	logMAR, final (left)	0.034	0.026
5254	Intra-ocular pressure, corneal-compensated (right)	0.125	0.138
5255	Intra-ocular pressure, Goldmann-correlated (right)	0.227	0.228

5256	Corneal hysteresis (right)	0.173	0.180
5257	Corneal resistance factor (right)	0.228	0.228
5262	Intra-ocular pressure, corneal-compensated (left)	0.139	0.141
5263	Intra-ocular pressure, Goldmann-correlated (left)	0.233	0.231
5264	Corneal hysteresis (left)	0.139	0.153
5265	Corneal resistance factor (left)	0.192	0.204
20015	Sitting height	0.444	0.451
20019	Speech-reception-threshold (SRT) estimate (left)	0.033	0.033
20021	Speech-reception-threshold (SRT) estimate (right)	0.034	0.033
20022	Birth weight	0.141	0.150
20127	Neuroticism score	0.133	0.133
20150	Forced expiratory volume in 1-second (FEV1), Best measure	0.207	0.207
20151	Forced vital capacity (FVC), Best measure	0.252	0.253
20161	Pack years of smoking	0.201	0.213
20162	Pack years adult smoking as proportion of life span exposed to smoking	0.248	0.263
21001	Body mass index (BMI)	0.282	0.284
21002	Weight	0.299	0.305
21021	Pulse wave Arterial Stiffness index	0.033	0.039
23099	Body fat percentage	0.298	0.300
23102	Whole body water mass	0.386	0.392
23106	Impedance of whole body	0.321	0.325
23107	Impedance of leg (right)	0.314	0.315
23108	Impedance of leg (left)	0.313	0.314
23109	Impedance of arm (right)	0.269	0.275
23110	Impedance of arm (left)	0.272	0.279
23111	Leg fat percentage (right)	0.311	0.311
23114	Leg predicted mass (right)	0.319	0.326
23115	Leg fat percentage (left)	0.306	0.307
23118	Leg predicted mass (left)	0.310	0.317
23119	Arm fat percentage (right)	0.279	0.282
23122	Arm predicted mass (right)	0.329	0.333
23123	Arm fat percentage (left)	0.281	0.285
23126	Arm predicted mass (left)	0.326	0.333
23127	Trunk fat percentage	0.278	0.281
23130	Trunk predicted mass	0.418	0.424
